# Circulating Tumor Cells as a Predictive Biomarker in Resectable Lung Cancer: A Systematic Review and Meta-Analysis

**DOI:** 10.3390/cancers14246112

**Published:** 2022-12-12

**Authors:** Durgesh Wankhede, Sandeep Grover, Paul Hofman

**Affiliations:** 1 Department of Surgical Oncology, All India Institute of Medical Sciences, New Delhi 110029, India; 2 Center for Human Genetics, Universitatsklinikum Giessen und Marburg—Standort Marburg, 35055 Marburg, Germany; 3 Laboratory of Clinical and Experimental Pathology, CHU Nice, FHU OncoAge, University Côte d’Azur, 06100 Nice, France; 4 Team 4, IRCAN, UMR 7284/U10181, FHU OncoAge, University Côte d’Azur, 06107 Nice, France; 5 Hospital-Integrated Biobank (BB-0033-00025), CHU Nice, FHU OncoAge, University Côte d’Azur, 06100 Nice, France; 6 European Liquid Biopsy Society, Martinistrasse 52 Building N27 Room 4.003, 20246 Hamburg, Germany

**Keywords:** lung cancer, non-small cell lung cancer, NSCLC, surgery, circulating tumor cells, prognosis

## Abstract

**Simple Summary:**

Lung cancer is the most common cause of cancer-related deaths worldwide. Circulating tumor cells (CTC) are cancer cells that are disseminated in the bloodstream and may be responsible for early recurrence and poor rates of survival. We conducted a systematic review and meta-analysis of 18 prospective studies comprising 1321 patients that reported CTC in resectable non-small cell lung cancer. Our analysis revealed that the presence of CTC in both the baseline and postoperative period was associated with an increased risk of recurrence and death compared to an absence of CTC in lung cancer patients. The results were independent of sources of CTC (peripheral, pulmonary vein), detection methods, and follow-up duration. CTCs hold a significant prognostic and predictive potential, as evident in our meta-analysis; however, heterogeneity of data, publication bias, and variable cut-off values limit its clinical utility.

**Abstract:**

Background: In breast, prostate, and other epithelial tumors, circulating tumor cells (CTC) in peripheral blood may predict survival. Our study evaluated the prognostic significance of baseline and postoperative CTC in patients with early non-small cell lung cancer (NSCLC) through a meta-analytic approach. Methods: Prospective studies comparing survival outcomes between positive (CTC+) and negative CTC (CTC−) patients were systematically searched. Primary outcomes were overall (OS) and disease-free survival (DFS) with hazard ratio (HR) and 95% confidence interval (CI) as the effect measure. Pooled HR determined the prognostic role under a fixed-effect or random-effect model depending on heterogeneity. Results: Eighteen studies with 1321 patients were eligible. CTC+ patients were associated with an increased risk of death (HR 3.53, 95% CI 2.51–4.95; *p* < 0.00001) and relapse (HR 2.97, 95% CI 2.08–4.22; *p* < 0.00001). Subgroup analysis results were consistent in different subsets, including time points (baseline and postoperative) and sources (peripheral and pulmonary vein) of blood collection, detection methods (label-free, label-dependent, and RT-PCR), and follow-up duration. Conclusion: Our meta-analysis revealed that CTC is a promising predictive biomarker for stratifying survival outcomes in patients with early-stage NSCLC. However, future studies are required to validate these findings and standardize detection methods.

## 1. Introduction

According to Global Cancer Statistics 2020, lung cancer accounts for one in ten new cases (11.4%) and one in five cancer-related mortalities (18.0%), making it the deadliest and the second most frequently diagnosed cancer [1]. Non-small cell lung cancer (NSCLC) represents 85% of new lung cancer cases and comprises adenocarcinoma (LUAD), squamous cell carcinoma (LSCC), and large-cell carcinoma (LCC) [2,3]. Early-stage NSCLC is seen in 40% of new cases and carries a 30–60% overall survival (OS) rate [4]. Despite curative treatment, around 30–55% of patients relapse, primarily at distant sites, and 50% succumb to lung cancer, suggesting the early dissemination of malignant cells [5]. Hence, it is imperative to explore effective strategies for evaluating the mortality and relapse risk in early-stage NSCLC.

Circulating tumor cells (CTC) get dislodged from the primary tumor or metastatic sites and circulate in the bloodstream [6]. However, most CTCs are cleared from the circulation, with only a small proportion achieving the potential to seed active metastatic tumors or remain dormant [disseminated tumor cells, (DTC)] to be activated later to form overt metastasis [7,8]. The dissemination of CTC may precede the formation of metastases and even primary tumors [9]. The entire genomic landscape of a patient’s tumor burden is etched in CTC, making it a commodity of great clinical significance [10]. Prompt identification of features of dissemination, such as CTC, provides an excellent opportunity for prognostic stratification and attuning of therapeutic modalities. 

The isolation and enrichment of CTC involve leveraging their biophysical properties (label independent), differential gene expression (label dependent), or a combination of both [11]. The CellSearch^®^ system (Veridex) was the first Food and Drug Administration (FDA)-approved device for enumerating epithelial-origin CTC. It incorporates EpCAM-coated magnetic nanoparticles for positive selection of EpCAM+ CTC, followed by immunofluorescence staining of Cytokeratin 8, 18, and 19 and an absence of CD45. CellSearch-detected CTC showed a prognostic association with metastatic breast, colorectal, and prostate cancer [12,13,14]. However, tumor cells exhibit phenotypic heterogeneity with differential expression of EpCAM according to tumor origin and stage [epithelial to mesenchymal (EMT) phenotype] and may even be absent during EMT and in non-epithelial tumors [15,16]. Despite the high CTC detection rates in NSCLC across all stages, not all studies have found a profound prognostic and/or predictive potential [17,18].

Prior meta-analysis on NSCLC showed that CTC positivity (CTC+) was associated with poor overall survival (OS) (RR = 2.19; 95% CI: 1.53–3.12; *p* < 0.0001) and disease-free survival (DFS) (RR = 2.14; 95% CI: 1.36–3.38; *p* < 0.0001) [19]. The preponderance of studies (15 of 20) with advanced stages in this meta-analysis left numerous questions unanswered. Firstly, CTC counts significantly increase in the pulmonary vein after surgical manipulation of the tumor or even endoscopic biopsy [20,21,22]. However, the prognostic implication of CTC+ in the pulmonary vein is variable, with some studies having shown a correlation between CTC and OS and progression-free survival (PFS) [23,24,25], while others have not [20,21,22,26]. Similarly, data on the predictive value of postoperative CTC are discordant [26,27]. Variable follow-up periods, numerous detection methods, inconsistent CTC cut-off values, and a relatively small patient cohort may influence the outcome. Resolution of these issues will aid in the bench-to-bedside transition of CTC in resectable NSCLCs. Therefore, we performed a meta-analysis of prospective studies to evaluate the prognostic role of baseline and postoperative CTC in resectable NSCLC.

## 2. Methods

The revised guidelines laid down by the statement protocol of Preferred Reporting Items for Systematic Review and Meta-analyses (PRISMA) were utilized to conduct this review (Tables S1 and S2) [28]. This review was not recorded on prospective registers; thus, a review protocol was not prospectively available. 

### 2.1. Data Sources and Search Strategy

A comprehensive and systematic literature search was conducted in PubMed, Embase, and Cochrane database. The following combinations of keywords and Medical Subject Headings (MeSH)/Emtree terms were used: “circulating tumor cells”, “circulating cancer cells”, “disseminated tumor cells”, “lung cancer”, and “non-small cell lung cancer” (Supplemental File).

### 2.2. Study Selection

Two independent reviewers (DW and SG) screened the titles and abstracts according to the subject’s relevance to determine full-text review eligibility. Full-text articles were scrutinized according to the inclusion and exclusion criteria. The publication that provided the most recent or informative data for studies with multiple publications was selected. Any difference in opinion regarding the final study eligibility was managed through mutual discussion.

The inclusion criteria were as follows: (1) studies assessing circulating tumor cells at baseline and/or postoperative period; (2) studies reporting time-to-event data, including OS and disease-free survival (DFS) for the individual groups of interest, i.e., CTC+ and absence of CTC (CTC−); (3) prospective studies including randomized and non-randomized clinical trials, and prospective observational studies; (4) studies published in English in a peer-reviewed journal from inception until June 2022.

Exclusion criteria were as follows: (1) retrospective studies, case reports, case series, abstracts, narrative and systematic reviews, and editorials; (2) advanced clinical stage (cIIIb-IV) or small-cell lung cancer (SCLC); and (3) utilization of ct-DNA or circulating exosomes for prognostic analysis. The PICO (population, intervention, comparison, and outcome) criteria for this study are presented in Table 1.

### 2.3. Data Abstraction

Two reviewers (DW and SG) independently extracted data from eligible studies. The following information was extracted: study characteristics (author, country, and year of publication), number of patients, demographics, histology, disease stage, treatment received (neoadjuvant and/or adjuvant), number of CTC+ patients according to time (baseline and postoperative), source (peripheral and pulmonary vein) of blood collection, and detection methods. CTC+ patients were defined as per the defined cut-offs of the detection method in the individual studies. In addition, HRs and associated confidence intervals (CIs) for OS and DFS were extracted. The period between the time of surgery and death from any cause was defined as OS, whereas the period between the time of surgery and cancer recurrence or metastasis was considered as DFS. We assessed whether the survival outcomes were adjusted for clinicopathological covariates through univariate or multivariate analysis. If the author reported univariate and multivariate survival analysis results, we would utilize the latter. In the absence of reported HR, Tierney’s or Parmar’s method was used to extract time-to-event data [29,30].

The Newcastle–Ottawa Scale (NOS) was used to assess the methodological quality of studies. It utilizes the star system in three categories: selection of the study population, comparability, and research outcome. The highest possible score is 9, and the score defines a study as low (7–9), moderate (4–6), or high risk (0–3) of bias [31].

### 2.4. Statistical Analysis

Pooled HR was used to evaluate the prognostic role of CTC for OS and DFS using the generic inverse variance method. The Cochran’s Q test was used to ascertain heterogeneity and was measured using the I^2^ index (0–100%). Low heterogeneity was defined as I^2^ less than 25%, moderate heterogeneity as I^2^ between 25% and 60%, and significant heterogeneity as I^2^ greater than 60% [32]. When heterogeneity was significant, the random-effects model was used; otherwise, the fixed-effects model was used. A p-value of less than 0.05 was considered statistically significant. A sensitivity analysis was performed to assess the validity of the results using the leave-one-out method. Begg’s funnel plots and Egger’s regression test were utilized to detect publication bias. In the case of publication bias, Duval and Tweedie’s trim-and-fill method was used to determine the adjusted summary effect (adjusted HR, (HR)) [33].

Subgroup analysis was performed according to the source (peripheral and pulmonary veins) of blood collection, detection methods (label-dependent, label-free, and RTPCR), and follow-up duration (>24 months). A 99% CI was used for the study estimates and a 95% CI for the summary estimates to decrease the likelihood of chance differences arising from multiple testing in the subgroup analyses. The association of clinical covariates [male sex, adenocarcinoma, and stage I-III] with CTC was determined by calculating pooled odds ratios (pORs) with 95% CIs for binary variables and differences in means (with SDs) for continuous variables [34,35]. The meta-analysis was performed using Review Manager version 5.4 (The Nordic Cochrane Center, The Cochrane Collaboration, Copenhagen, Denmark), whereas publication bias was assessed in the JASP software (JASP 0.15, the JASP team) [36].

## 3. Results

**Figure 1:**
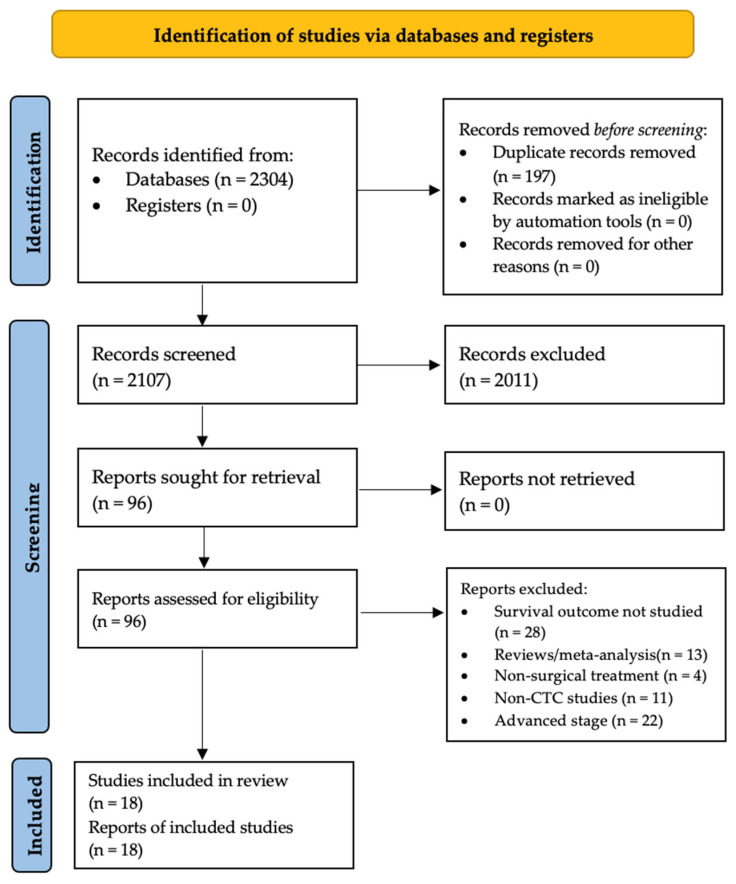
Flow diagram of study selection. The flow diagram is presented according to the Preferred Reporting Items for Systematic Reviews and Meta-Analyses (PRISMA) guidelines.

The PRISMA flow diagram of the study selection is shown in Figure 1. A preliminary search of titles and abstracts yielded 2304 articles, of which 1897 were removed because of irrelevance. The remaining articles underwent a full-text review and were scrutinized according to the inclusion and exclusion criteria. Consequently, 18 (4.1%) [24,25,26,37,38,39,40,41,42,43,44,45,46,47,48,49,50,51] studies were included in the analysis.

### 3.1. Study Characteristics

Detailed study characteristics are summarized in Table 2. In the 18 eligible studies, the total number of included patients was 1321, ranging from 23 to 208 patients per study (median: 65). Most studies were published between 2010 and 2021. Eight studies were conducted in Europe, nine in Asia, and one in North America. The median age ranged from 59–68 years, and 63% and 83% of the patients were male and ever smokers, respectively. Adenocarcinoma was the predominant histology. Pathologic stages I, II, and III were observed in 44%, 23%, and 23% of the pooled study population. Two studies used neoadjuvant therapy in their treatment protocol, and 37% of the pooled study population received adjuvant therapy. The median follow-up range was 14–84 months. 

Label-dependent detection was used in seven studies, of which CellSearch^®^ and MACS systems were used in three studies each, whereas one used the CytoploRare^®^ platform. The label-free method was utilized in seven studies using the following platforms: ScreenCell^®^ (n = 1), CanPatrol^®^ (n = 1), Ficoll-Plaque^TM^ (n = 2), ISET^®^ (n = 1), CellSieve ^®^ (n = 1), and microfilter technique (n = 1). The remaining studies utilized RT-PCR. The collected blood volume ranged between 1–20 mL, and the most frequently drawn volume was 7.5 mL (n = 3), while two studies did not report the volume.

### 3.2. Quality of the Included Studies

The Newcastle–Ottawa quality assessment scale for cohort studies was used to assess the methodological quality of studies. The study quality scores from the NOS system are summarized in Supplementary Table S3. Eleven (64.7%) of eighteen studies were identified as having a low risk of bias, five as moderate, and two as high-risk. The median score was 7, while the mean score was 6.5, indicating moderate-to-high methodological quality.

### 3.3. Meta-Analysis

#### 3.3.1. Primary Analysis

**Figure 2:**
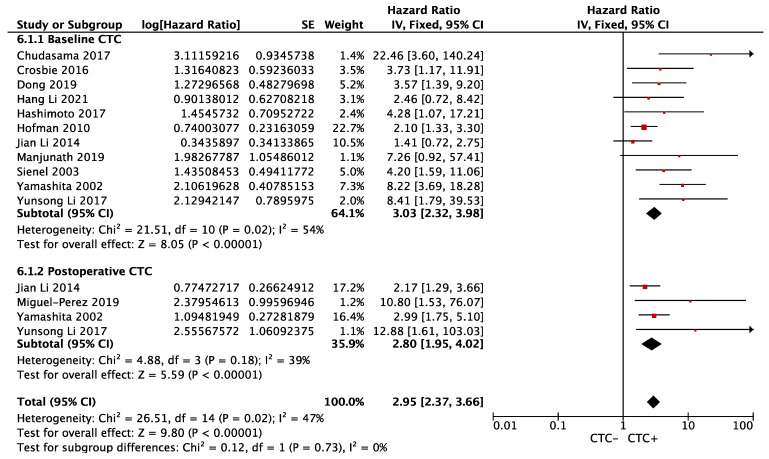
Effect of baseline or postoperative CTC on overall survival in resectable NSCLC. Hazard ratios with 95% confidence intervals are displayed by individual studies, describing pooled overall effects for baseline CTC and postoperative CTC, respectively. Abbreviations: HR, hazard ratio; SE, standard error; CI, confidence interval.

Eleven (preoperative = 7, postoperative = 4) and fourteen (preoperative = 9, postoperative = 5) studies were included in OS and DFS analyses, respectively. The meta-analysis revealed that CTC+ was associated with an increased risk of death (Overall HR 2.95, 95% CI 2.37–3.66; *p* < 0.00001), regardless of baseline (HR 3.03, 95% CI 2.32–3.98; *p* < 0.00001, I^2^ = 54%) and postoperative period (HR 2.80, 95% CI 1.95–4.02; *p* < 0.00001, I^2^ = 39%). There was no subgroup difference (*p* = 0.62, I^2^ = 0%), and this was associated with moderate heterogeneity (p_het_ = 0.02, I^2^ = 47%) (Figure 2). Sensitivity analysis showed a stable result.

**Figure 3:**
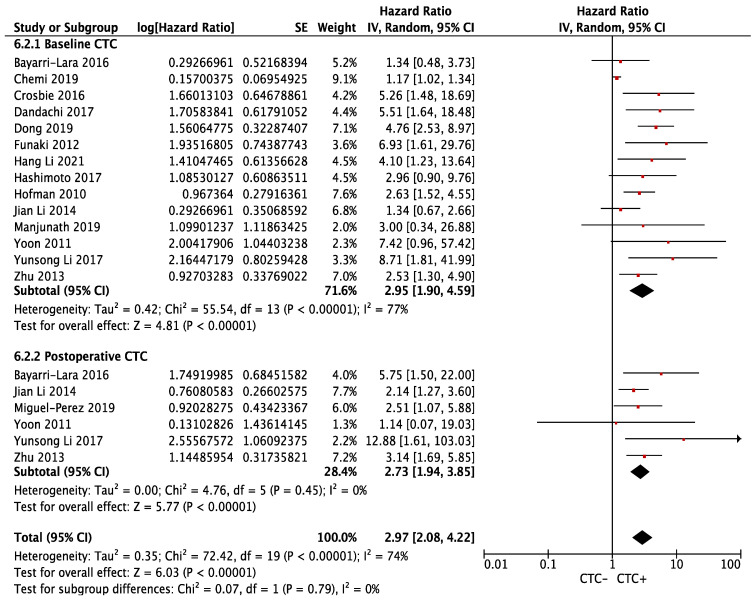
Effect of baseline or postoperative CTC on disease-free survival in resectable NSCLC. Hazard ratios with 95% confidence intervals are displayed by individual studies, describing pooled overall effects for baseline CTC and postoperative CTC, respectively. Abbreviations: HR, hazard ratio; SE, standard error; CI, confidence interval.

Similarly, the risk of relapse was significantly higher in the CTC+ group (overall HR 2.97, 95% CI 2.08–4.22; *p* < 0.00001), regardless of baseline (HR 2.95, 95% CI 1.90–4.59; *p* < 0.00001, I^2^ = 77%) or postoperative blood collection (HR 2.73, 95% CI 1.94–3.85; *p* < 0.00001, I^2^ = 0%). The result showed significant heterogeneity (*p*_het_ < 0.00001, I^2^ = 74%) (Figure 3). Sensitivity analysis revealed that Chemi et al. (baseline CTC subgroup) contributed significantly to the heterogeneity, which, when removed, led to an increased pooled HR for baseline CTC (HR 3.09, 95% CI 2.25–4.25; *p* < 0.00001, I^2^ = 25%, p_het_ = 0.19) (Supplemental Figure S1).

#### 3.3.2. Subgroup Analyses

The results of subgroup analysis were consistent for both peripheral sources (OS: HR 2.99, 95% CI 1.78–5.03; *p* < 0.0001, I^2^ = 51%; DFS: HR 2.77, 95% CI 1.88–4.08; *p* < 0.00001, I^2^ = 32%) and pulmonary venous source (OS: HR 2.68, 95% CI 1.24–5.77; *p* = 0.01, I^2^ = 72%; DFS: HR 2.16, 95% CI 1.82–3.66; *p* = 0.002, I^2^ = 85%) of blood collection (Supplemental Figure S2). 

Furthermore, the subgroup analysis based on detection methods revealed that CTC+ detected by all (label dependent: HR 3.95 95% CI 2.06–7.60; *p* < 0.0001, I^2^ = 0%; label-free: 3.91 (95% CI 2.01–7.62; *p* < 0.0001, I^2^ = 52%) except RTPCR (HR 3.36, 95% CI 0.60–18.88; *p* = 0.17) showed a negative correlation with OS. On the other hand, all three subgroups showed that CTC+ detected by any method is associated with an increased risk of relapse (label dependent: HR 2.62, 95% CI 1.28–5.34; *p* = 0.004, I^2^ = 71%; label-free: HR 3.71, 95% CI 2.55–5.38; *p* < 0.00001, I^2^ = 0%; and RTPCR: HR 2.11, 95% CI 1.08–4.12; *p* = 0.03, I^2^ = 40%) (Supplemental Figure S3). Similarly, the results were congruous regardless of the duration of follow-up (<24 months (OS: HR 2.37, 95% CI 1.57–3.59; *p* < 0.0001, I^2^ = 0%; DFS: HR 3.06, 95% CI 1.95–4.79; *p* < 0.00001, I^2^ = 11%; >24 months (OS: HR 3.64, 95% CI 2.55–5.20; *p* < 0.00001, I^2^ = 59%; DFS: HR 2.75, 95% CI 1.54–4.93; *p* = 0.0007, I^2^ = 80%) (Supplemental Figure S4).

#### 3.3.3. Publication Bias

The regression tests for funnel plot asymmetry (“Egger’s test”) for the primary analyses (OS and DFS) were statistically significant (*p* < 0.001) (Supplemental Figure S5). The trim-and-fill analysis for the OS outcome led to adding seven studies, with an adjusted summary estimate (HR) of 2.48 (95% CI 1.66–3.70). Similarly, seven studies were filled for the DFS outcome, which led to an adjusted pooled HR of 2.24 (95% CI 1.71–2.97) (Supplemental Figure S6). The outcome estimate remained statistically significant after adjusting for publication bias, suggesting robustness of our results. 

#### 3.3.4. Association of CTC with Clinicopathologic Factors

Male patients (OR 1.02, 95% CI 0.78–1.35 *p* = 0.87) and adenocarcinoma (OR 1.30, 95% CI 0.76–2.23 *p* = 0.33) were not associated with CTC+ status. Pathologic stage showed a stage-wise progression of association with CTC, with stage I (OR 0.62, 95% CI 0.42–0.93; *p* = 0.02) having the least likelihood to greatest chance in stage III (OR 2.65, 95% CI 1.51–4.64; *p* = 0.0007). Stage II showed no association with CTC+ status (OR 1.30, 95% CI 0.83–2.04 *p* = 0.24) (Supplemental Figure S7).

## 4. Discussion

In the present study, a systematic review and meta-analysis of eighteen prospective studies was conducted, evaluating the prognostic significance of CTC in baseline and postoperative samples from patients with early-stage NSCLC. Meta-analytic data showed that CTC status was highly predictive of the survival outcomes of patients with early-stage NSCLC. Specifically, CTC+ status had a negative impact on OS and DFS, regardless of time and source of blood collection, detection methods, and follow-up duration. In addition, we found that the pathological stage was associated with CTC status, with stage III patients more likely to be CTC+, whereas stage I was at a lower risk. Significant heterogeneity was observed among the included studies; however, sensitivity analyses revealed stable results. Our findings are consistent with the growing body of evidence that demonstrates that CTCs are promising prognostic markers for resectable cancers and resolve various issues revolving around the bench-to-bedside transition of CTC in early-stage NSCLC [52,53,54,55].

Prior meta-analyses have reported on the prognostic significance of CTC in lung cancer patients and suggested that CTC+ status was associated with adverse survival outcomes, which is consistent with our results [19,56,57,58]. However, their methodology differed significantly from the present study, and some of their results required further improvement. Firstly, the selection criteria were not precise. Some meta-analyses included studies with varying histopathological subtypes, such as lung cancer (NSCLC and SCLC) or only SCLC, whereas others included both early and advanced-stage NSCLC [19,56,58]. A common thread among these studies was the presence of heterogenous populations, including a significant proportion of advanced or metastatic lung cancer patients who underwent treatment modalities that were non-curative in intent. Thus, the data on the role of CTC on OS and PFS were available; however, its association with DFS remained unknown. Secondly, until now, CTCs were not utilized to monitor treatment response following surgical therapy for lung cancer. Five of the eight studies used in an earlier meta-analysis to determine whether post-treatment CTCs were predictive had chemoradiotherapy as the primary therapy [56]. Therefore, we exclusively incorporated patients undergoing curative resection and succeeded in showing the pooled prognostic effect of CTC in early-stage NSCLC.

The most proximal venous channel to the primary tumor is likely to encounter the highest burden of CTC, thereby making the pulmonary vein a promising area for CTC catchment, considering the rarity of CTC in peripheral blood [59]. The pulmonary vein CTC detection rate was higher (almost 100%) and had considerably higher CTC counts than the peripheral blood source [20,21,23,24]. Moreover, intraoperative tumor manipulation may initially release CTC in the pulmonary circulation; however, eventually, they are cleared by shear stress or immune reaction [60,61]. According to recent studies, ligating the pulmonary vein before the artery and implementing a “no-touch technique” are two of the most effective ways to prevent tumor cell dissemination [62,63]. However, the mere presence of CTC does not equate to the subsequent risk of metastasis and reflects the true tumor burden. Approximately 67% of pulmonary vein CTC are misidentified and found to have no genomic aberrations and are, in fact, epithelial cells [64]. Xenograft assays have shown that only 33% of CTC have the tumorigenic potential [21]. It can be hypothesized that innumerable epithelial cells are released into the circulation from the primary tumor, albeit only a small percentage develop the potential to be malignant and affect survival outcomes [65]. Our results revealed that the pooled survival outcome for CTC detected from the pulmonary vein to the peripheral source were similar, strengthening this hypothesis. 

In addition to phenotypic identification, tumor genomic profiling of CTC is another promising avenue for prognostic exploration. Depending upon EMT markers, CTC are classified into epithelial (E-CTC), expressing markers like EpCAM and CK, mesenchymal (M-CTC), expressing vimentin and twist, and bi-phenotype/hybrid CTC [66]. Different CTC subpopulations may exhibit differential tumor characteristics and survival outcomes based on their phenotype. E-CTC+ is associated with localized disease, although it correlates with a shorter OS [67]. Furthermore, patients with a preponderance of mesenchymal CTC (M-CTC) and hybrid CTC were negatively correlated with postoperative DFS, OS, and therapeutic response [9,68,69]. Detection methods come into play when label-dependent devices such as CellSearch^®^ show a divergent CTC profile. CellSearch^®^ failed to detect CTC in early-stage NSCLC, with only a 22–25% detection rate for metastatic disease [18,70]. Hofman et al. suggested that label-free and label-dependent methods are complementary rather than competitive, with a more robust survival outcome using both methods than just one [71]. Our subgroup analysis showed that CTC detected by any method correlated with inferior OS and DFS; the label-free method showed a considerably worse DFS (HR 3.71; *p* < 0.00001) than the other methods. Although it was not possible to comment on the prognostic role of specific phenotypes, our data indicate that detection methods may not influence survival outcomes. More studies are required to validate this finding.

Clinical predictors of the presence of CTC may allow for an indirect method of ascertaining prognosis. We found that sex and histology were not associated with CTC, contrary to other studies [72,73]. An interesting pattern emerged during the stage assessment. As the stage progressed from early to advanced, the likelihood of CTC+ increased accordingly. Stage I disease was least likely to be associated with CTC resembling the classical view of the impact of the stage on CTC. Moreover, CTC count correlated with the pathologic stage in a similar way [74,75]. These findings hint towards the increased shedding of CTC from the bulky tumor and multiple nodal metastases. Moreover, this result also conforms to the current evidence on the association of CTC to tumor size and lymph node metastasis [76,77].

The present study has a number of strengths. Due to the inclusion of prospective clinical studies exclusively, the limitation and bias of retrospective studies were avoided, thereby increasing the quality of evidence. Moreover, consistent results on sensitivity and subgroup analyses and a moderate to high methodological quality of individual studies further support the robustness of our results. For the first time, our study demonstrated the pooled prognostic effect of CTC in terms of DFS in resectable NSCLC.

Despite resolving various contentions regarding the clinical utility of CTC, certain limitations prevailed. Firstly, non-English language studies were excluded leading to language bias that may influence the summary effects [78]. Secondly, the effects of unmatched confounders could not be adjusted due to non-randomized baseline characteristics and the derivation of effect estimates from extracted data or univariate analysis. Thirdly, summary study-level data were used in this meta-analysis, which have lower statistical power than individual patient-level data [79,80]. Fourthly, CTC detected by RT-PCR failed to correlate with OS, possibly due to fewer studies (n = 2) and participants. In addition, the presence of significant heterogeneity indicates that the validity of the treatment effect estimate in the RT-PCR subgroup is uncertain. Moreover, we could not comment on the effect of CTC subpopulations on the prognosis. Cut-off values may exert a significant influence on the pooled estimates and heterogeneity. However, we failed to assess the same because of the lack of data. It may also contribute to data availability bias [81]. Lastly, an apparent publication bias existed in our results, which, when adjusted, decreased the pooled estimate for OS and DFS.

Currently, tumor genomic profiling through liquid biopsy is predominated by circulating tumor DNA (ctDNA) analysis because of its relative ease of extraction and comparatively lower cost than CTC analysis. Studies have shown that ctDNA is predictive of necrosis, lymphovascular invasion, a high proliferative index, tumor stage, and nodal metastasis in surgically resected NSCLC [65,82]. There has been a significant development in the use of CTCs and ctDNAs in the determination of minimal residual disease (MRD), which is the post-treatment tumor burden that cannot be detected by conventional diagnostic methods [83]. Discordant mutation profiles between plasma and tissue samples, between ctDNA sources, and potential false-positive findings limit the widespread clinical utility of ctDNA in determining MRD [65,83,84].

On the other hand, CTC analysis provides several advantages over ctDNA analysis, such as identifying protein biomarkers and tumor heterogeneity, detecting driver mutations in subclonal neoplastic cells, and studying the evolution of tumor pathogenesis and dissemination [85]. A prospective study on CTC-based MRD detection in early-stage NSCLC patients following curative surgery has shown that an increase in CTCs on postoperative days 1 and 3 correlated with early relapse, opening doors for microscopic relapse surveillance in the future [27]. In addition, CTCs are detected in all patients with lung cancer, unlike ctDNA, which is detected in only 50% of stage I patients [86,87]. Ultimately, CTC, but not DNA, has been proven to seed distant metastasis [88].

An inclusive approach in liquid biopsy by complementing CTC analysis with ct-DNA or incorporating different detection methods simultaneously may augment our understanding of the tumor status of the patient. Further validation of the clinical utility of CTC requires standardizing detection methods and designing randomized intervention trials to tailor therapeutic decisions to CTC analysis.

## 5. Conclusions

Our meta-analysis based on eighteen prospective clinical studies revealed that CTC+ status was associated with poor OS and DFS in surgically resected NSCLC patients, regardless of the time or source of blood collection, detection methods, or follow-up duration. Although this meta-analysis was undermined by significant heterogeneity and publication bias, our findings suggest that CTC status might be a promising predictive biomarker of mortality and recurrence in early-stage NSCLC. This study takes us one step closer to CTC’s bench-to-bedside transition and facilitates microscopic relapse surveillance in early-stage lung cancer.

## Figures and Tables

**Table 1:** PICO framework.

**Participants**	Adult patients with histologically confirmed, resectable NSCLC (Clinical Stage I-IIIA)
**Intervention**	Curative lung resection (lobectomy/pneumonectomy/wedge resection/segmentectomy with mediastinal lymph node dissection/sampling)
**Comparison**	Presence of CTC (CTC+) versus absence of CTC (CTC−)
**Outcome**	Overall survival and disease-free survival

**Table 2:** Clinical characteristics.

Study	Study Design	Study Period	Patients (n)	Age (Median, Years)	Male (n, %)	Smokers (n, %)	Histology (n)		Pathologic Stage (n)	Follow-Up (Median, Months)	CTC Present (n)		Detection Method		NOS Score
							ADC	SCC	I/II/III		Base Line	Postoperative	Category	Device	
Bayarri-Lara 2016, Spain	Prospective longitudinal cohort	2012–2014	56	67	50 (89)	53 (95)	25	29	26/22/8	16	29	18	Label-dependent	MACS	8
Chemi 2019, UK	Multicenter, prospective cohort	2014–2017	100	68	61 (61)	92 (92)	59	41	47/34/19	32.6	48	NI	Label-dependent	CellSearch	9
Chudasama 2017, UK	Prospective cohort	2014–2014	23	66	9 (39)	NA	13	10	18 */18 */5	31.8	18	NI	Label-free	ScreenCell	5
Crosbie 2016, UK	Prospective cohort	NA	30	67	16 (53)	30 (100)	8	21	12/11/7	22	13	NI	Label-dependent	CellSearch	8
Dandachi 2017, Austria	Prospective cohort	2015–2016	40	67	16 (40)	27 (67)	40	0	19/9/12	15.8	15	NI	Label-free	Microfilter	6
Dong 2019, China	Prospective cohort	2016–2018	114	59	65 (57)	NA	83	28	51/21/42	30	110	NI	Label-free	CanPatrol	7
Funaki 2012, Japan	Prospective cohort	2008–2010	130	68	56 (43)	NA	92	26	98/20/12	19	53	NI	Label-free	Ficoll-Paque-Plus	3
Hashimoto 2017, Japan	Prospective cohort	2009–2010	30	68	18 (60)	NA	22	6	17/13 */13 *	64.4	24	NI	Label-dependent	CellSearch	7
Hofman 2010,France	Prospective cohort	2006–2009	208	63	141 (68)	189 (91)	115	54	86/51/58	24	102	NI	Label-free	ISET	6
Li Jian 2014, China	Prospective cohort	2007–2009	68	63	47 (69)	NA	44	22	16/36/16	39.5	40	22	RT-PCR	qRTPCR: LUNX mRNA	7
Li Yunsong 2017, China	Prospective cohort	2010–2010	23	61	8 (35)	NA	11	12	8/7/8	60	10	6	Label-dependent	MACS	6
Li Hang 2021, China	Prospective cohort	2012–2012	54	61	30 (55)	NA	38	11	26/26 */26 *	84	14	NI	Label-dependent	Cytoplo-Rare	7
Manjunath 2019, USA	Prospective clinical trial	2016–2018	30	65	16 (53)	0	18	10	16/8/6	14.3	30	NI	Label-free	CellSieve	7
Miguel-Perez 2019, Spain	Prospective longitudinal cohort	2012–2015	97	66	84 (86)	88 (91)	47	50	44/25/18	30.5	40	27	Label-dependent	MACS	8
Sienel 2003, Germany	Prospective cohort	1996–2001	62	62	45 (72)	NA	19	28	NA	25	11	NI	Label-free	Ficoll-Paque	3
Yamashita 2002, Japan	Prospective cohort	1996–1998	103	68	76(74)	NA	66	37	57/19/27	35	29	27	RT-PCR	RTPCR for CEA mRNA	5
Yoon 2011, South Korea	Prospective longitudinal cohort	2007–2008	79	66	48 (60)	NA	45	27	45/19/15	60	26	12	RT-PCR	RTPCR for CK19, TTF-1	7
Zhu 2013, China	Prospective cohort	2008–2012	74	63	49 (66)	NA	41	25	15/28/22	32	4	16	RT-PCR	qRTPCR of EpCAM and MUC1	8

*: not reported separately. ADC: adenocarcinoma, SCC: squamous cell carcinoma, CTC: circulating tumor cells, MACS: magnetic-activated cell sorting, NA: not available, NI: not included, UK: United Kingdom, ISET: isolation by size of tumor cells, RT-PCR: reverse transcriptase PCR, NOS: Newcastle–Ottawa Scale.

## Supplementary Materials

The following supporting information can be downloaded at: https://www.mdpi.com/article/10.3390/cancers14246112/s1, Table S1. PRISMA abstract checklist, Table S2. PRISMA 2020 checklist, Table S3. Study quality score, Figure S1. Sensitivity analysis: Correlation of CTC with Disease-free survival (Chemi et al. excluded), Figure S2. Subgroup analysis: Source of blood collection, Figure S3. Subgroup analysis: Detection methods, Figure S4. Subgroup analysis: Duration of follow-up, Figure S5. Publication bias: Funnel plot for primary analysis, Figure S6. Publication bias: Trim-and-fill method, Figure S7. Correlation of CTC with clinicopathologic factors.
