# Supplementary material for: Circulating Tumor Cells as a Predictive Biomarker in Resectable Lung Cancer: A Systematic Review and Meta-Analysis
Source: Cancers (Basel). 2022 Dec 12;14(24):6112. doi: 10.3390/cancers14246112 (PMC9776809; doi:10.3390/cancers14246112)

Supplementary Material

Supplementary Methods

Search strategies on Medline, EMBASE and Cochrane Database

Characteristics of the excluded study

Supplementary Tables

Table S1. PRISMA abstract checklist

Table S2. PRISMA 2020 checklist

Table S3. Study quality score

Supplementary Figure

Figure S1. Sensitivity analysis: Correlation of CTC with Disease-free survival (Chemi et al. excluded)

Figure S2. Subgroup analysis: Source of blood collection

Figure S3. Subgroup analysis: Detection methods

Figure S4. Subgroup analysis: Duration of follow-up

Figure S5. Publication bias : Funnel plot for primary analysis

Figure S6. Publication bias: Trim-and-fill method

Figure S7. Correlation of CTC with clinicopathologic factors

Search strategies

	Pubmed			EMBASE			Cochrane Library	
1	Lung cancer	33971	1	Lung cancer	54317	1	Lung cancer	4025
2	Lung cancers	2976	2	Lung cancers	4180	2	Lung cancers	460
3	Lung carcinoma	7642	3	Lung carcinoma	7951	3	Lung carcinoma	715
4	Pulmonary carcinoma	1209	4	Pulmonary carcinoma	1406	4	Pulmonary carcinoma	159
5	Lung adenocarcinoma	5977	5	Lung adenocarcinoma	11035	5	Lung adenocarcinoma	1424
6	Lung adenocarcinomas	1001	6	Lung adenocarcinomas	1459	6	Lung adenocarcinomas	85
7	Lung squamous cell carcinoma	3442	7	Lung squamous cell carcinoma	11850	7	Lung squamous cell carcinoma	376
8	Non-small cell lung cancer	7486	8	Non-small cell lung cancer	1106	8	Non-small cell lung cancer	305
9	NSCLC	8651	9	NSCLC	9723	9	NSCLC	199
10.	#1 OR #2 OR #3 OR #4 OR #5 OR #6 OR #7 OR #8 OR #9	56245	10.	#1 OR #2 OR #3 OR #4 OR #5 OR #6 OR #7 OR #8 OR #9	71904	10.	#1 OR #2 OR #3 OR #4 OR #5 OR #6 OR #7 OR #8 OR #9	3019
11	Liquid Biopsy	2357	11	Liquid Biopsy	4159	11	Liquid Biopsy	514
12	Circulating tumor cells	1205	12	Circulating tumor cells	1672	12	Circulating tumor cells	1905
13	Circulating neoplastic cells	863	13	Circulating neoplastic cells	436	13	Circulating neoplastic cells	151
14	CTC	901	14	CTC	1657	14	CTC	573
15	#11 OR #12 OR #13 OR # 14	5083	15	#11 OR #12 OR #13 OR # 14	7351	15	#11 OR #12 OR #13 OR # 14	3418
16	#10 AND #15	2781	16	#10 AND #15	5073	16	#10 AND #15	215
						17	#16 with “trials”	86

Characteristics of the Excluded Studies

No.	Title	Reason for exclusion
1	Abbosh C, Birkbak NJ, Wilson GA, et al. Phylogenetic ctDNA analysis depicts early-stage lung cancer evolution. Nature. 2017;545(7655):446-451. doi:10.1038/nature22364	ct-DNA
2	Abbosh C, Birkbak NJ, Swanton C. Early stage NSCLC - challenges to implementing ctDNA-based screening and MRD detection. Nat Rev Clin Oncol. 2018 Sep;15(9):577-586. doi: 10.1038/s41571-018-0058-3. PMID: 29968853.	Ct-DNA, review
3	Torres J.A. (2021) Circulating Tumor Cells in the context Non-small Cell Lung Cancer. In: Chinen L.T.D. (eds) Atlas of Liquid Biopsy. Springer, Cham. https://doi.org/10.1007/978-3-030-69879-9_5	Review
4	Buscail E, Chiche L, Laurent C, et al. Tumor-proximal liquid biopsy to improve diagnostic and prognostic performances of circulating tumor cells. Mol Oncol. 2019;13(9):1811-1826. doi:10.1002/1878-0261.12534	Review
5	Lv C, Zhao B, Wang L, et al. Detection of circulating tumor cells in pulmonary venous blood for resectable non-small cell lung cancer. Oncol Lett. 2018;15(1):1103-1112. doi:10.3892/ol.2017.7405	Survival data not studied
6	Chaudhuri AA, Chabon JJ, Lovejoy AF, et al. Early Detection of Molecular Residual Disease in Localized Lung Cancer by Circulating Tumor DNA Profiling. Cancer Discov. 2017;7(12):1394-1403. doi:10.1158/2159-8290.CD-17-0716	ctDNA
7	Chemi F, Mohan S, Guevara T, Clipson A, Rothwell DG and Dive C (2021) Early Dissemination of Circulating Tumor Cells: Biological and Clinical Insights. Front. Oncol. 11:672195. doi: 10.3389/fonc.2021.672195	Review
8	Chaudhuri AA, Chabon JJ, Lovejoy AF, et al. Early Detection of Molecular Residual Disease in Localized Lung Cancer by Circulating Tumor DNA Profiling. Cancer Discov. 2017;7(12):1394-1403. doi:10.1158/2159-8290.CD-17-0716	Non-surgical treatment
9	Chen X, Wang X, He H, Liu Z, Hu J-F, Li W (2015) Combination of Circulating Tumor Cells with Serum Carcinoembryonic Antigen Enhances Clinical Prediction of Non-Small Cell Lung Cancer. PLoS ONE 10(5): e0126276. doi:10.1371/journal.pone.0126276	Advanced stage
10	Chudasama D, Burnside N, Beeson J, Karteris E, Rice A, Anikin V. Perioperative detection of circulating tumour cells in patients with lung cancer. Oncol Lett. 2017 Aug;14(2):1281-1286. doi: 10.3892/ol.2017.6366. Epub 2017 Jun 9. PMID: 28789342; PMCID: PMC5529936.	Survival outcome not studied
11	Dong J, Zhu D, Tang X, Qiu X, Lu D, Li B, Lin D and Zhou Q (2019) Detection of Circulating Tumor Cell Molecular Subtype in Pulmonary Vein Predicting Prognosis of Stage I–III Non-small Cell Lung Cancer Patients. Front. Oncol. 9:1139. doi: 10.3389/fonc.2019.01139	Overlapping study population
12	Duan X, Zhu Y, Cui Y, et al. Circulating tumor cells in the pulmonary vein increase significantly after lobectomy: A prospective observational study. Thorac Cancer. 2019;10(2):163-169. doi:https://doi.org/10.1111/1759-7714.12925	Survival outcome not studied
13	Fiorelli A, Accardo M, Carelli E, Angioletti D, Santini M, Di Domenico M. Circulating Tumor Cells in Diagnosing Lung Cancer: Clinical and Morphologic Analysis. Ann Thorac Surg. 2015 Jun;99(6):1899-905. doi: 10.1016/j.athoracsur.2014.11.049. Epub 2015 Feb 10. PMID: 25678504.	Survival outcome not studied
14	Funaki S, Sawabata N, Nakagiri T, Shintani Y, Inoue M, Kadota Y, Minami M, Okumura M. Novel approach for detection of isolated tumor cells in pulmonary vein using negative selection method: morphological classification and clinical implications. Eur J Cardiothorac Surg. 2011 Aug;40(2):322-7. doi: 10.1016/j.ejcts.2010.11.029. Epub 2011 Jan 7. PMID: 21215651.	Overlapping study population
15	Gallo M, Luca A De, Maiello MR, et al. Clinical utility of circulating tumor cells in patients with non-small-cell lung cancer. Transl Lung Cancer Res Vol 6, No 4 (August 2017) Transl Lung Cancer Res (Current Knowl Futur Perspect Circ Tumor Cells Lung Cancer). Published online 2017. https://tlcr.amegroups.com/article/view/13934	Review

16	Gao W, Yuan H, Jing F, et al. Analysis of circulating tumor cells from lung cancer patients with multiple biomarkers using high-performance size-based microfluidic chip. Oncotarget. 2017;8(8):12917-12928. doi:10.18632/oncotarget.14203	Survival outcome not studied
17	Ge MJ, Shi D, Wu QC, Wang M, Li L Bin. Observation of circulating tumour cells in patients with non-small cell lung cancer by real-time fluorescent quantitative reverse transcriptase-polymerase chain reaction in peroperative period. J Cancer Res Clin Oncol. 2006;132(4):248-256. doi:10.1007/s00432-005-0059-3	Survival outcome not studied
18	Huang HB, Ge MJ. The Effects of Different Surgical Approaches on the Perioperative Level of Circulating Tumor Cells in Patients with Non-Small Cell Lung Cancer. Thorac Cardiovasc Surg. 2016;64(06):515-519.	Survival outcome not studied
19	Habli Z, AlChamaa W, Saab R, Kadara H, Khraiche ML. Circulating Tumor Cell Detection Technologies and Clinical Utility: Challenges and Opportunities. Cancers . 2020;12(7). doi:10.3390/cancers12071930	Review
20	Hanssen A, Wagner J, Gorges TM, et al. Characterization of different CTC subpopulations in non-small cell lung cancer. Sci Rep. 2016;6(1):28010. doi:10.1038/srep28010	Advanced stage
21	Hashimoto M, Tanaka F, Yoneda K, et al. Significant increase in circulating tumour cells in pulmonary venous blood during surgical manipulation in patients with primary lung cancer †. Interact Cardiovasc Thorac Surg. 2014;18(6):775-783. doi:10.1093/icvts/ivu048	Survival outcome not studied
22	He Y, Shi J, Schmidt B, Liu Q, Shi G, Xu X, Liu C, Gao Z, Guo T, Shan B. Circulating Tumor Cells as a Biomarker to Assist Molecular Diagnosis for Early Stage Non-Small Cell Lung Cancer. *Cancer Manag Res*. 2020;12:841-854 https://doi.org/10.2147/CMAR.S240773	Survival outcome not studied
23	Hofman V, Long E, Ilie M, Bonnetaud C, Vignaud JM, Fléjou JF, Lantuejoul S, Piaton E, Mourad N, Butori C, Selva E, Marquette CH, Poudenx M, Sibon S, Kelhef S, Vénissac N, Jais JP, Mouroux J, Molina TJ, Vielh P, Hofman P. Morphological analysis of circulating tumour cells in patients undergoing surgery for non-small cell lung carcinoma using the isolation by size of epithelial tumour cell (ISET) method. Cytopathology. 2012 Feb;23(1):30-8. doi: 10.1111/j.1365-2303.2010.00835.x. Epub 2011 Jan 6. PMID: 21210876.	Survival outcome not studied, Overlapping study population
24	Hofman VJ, Ilie MI, Bonnetaud C, Selva E, Long E, Molina T, Vignaud JM, Fléjou JF, Lantuejoul S, Piaton E, Butori C, Mourad N, Poudenx M, Bahadoran P, Sibon S, Guevara N, Santini J, Vénissac N, Mouroux J, Vielh P, Hofman PM. Cytopathologic detection of circulating tumor cells using the isolation by size of epithelial tumor cell method: promises and pitfalls. Am J Clin Pathol. 2011 Jan;135(1):146-56. doi: 10.1309/AJCP9X8OZBEIQVVI. PMID: 21173137.	Survival outcome not studied, Overlapping study population
25	Hofman V, Ilie MI, Long E, et al. Detection of circulating tumor cells as a prognostic factor in patients undergoing radical surgery for non-small-cell lung carcinoma: comparison of the efficacy of the CellSearch AssayTM and the isolation by size of epithelial tumor cell method. Int J Cancer. 2011;129(7):1651-1660. doi:https://doi.org/10.1002/ijc.25819	Overlapping study population
26	Hosokawa M, Kenmotsu H, Koh Y, Yoshino T, Yoshikawa T, et al. (2013) Size-Based Isolation of Circulating Tumor Cells in Lung Cancer Patients Using a Microcavity Array System. PLoS ONE 8(6): e67466. doi:10.1371/journal.pone.0067466	Advanced stage, Survival outcome not studied
27	Huang J, Wang K, Xu J, Huang J, Zhang T (2013) Prognostic Significance of Circulating Tumor Cells in Non-Small-Cell Lung Cancer Patients: A Meta-Analysis. PLoS ONE 8(11): e78070. doi:10.1371/journal.pone.0078070	Meta-analysis
28	Rolfo C, Mack P, Scagliotti GV, Aggarwal C, Arcila ME, Barlesi F, Bivona T, Diehn M, Dive C, Dziadziuszko R, Leighl N, Malapelle U, Mok T, Peled N, Raez LE, Sequist L, Sholl L, Swanton C, Abbosh C, Tan D, Wakelee H, Wistuba I, Bunn R, Freeman-Daily J, Wynes M, Belani C, Mitsudomi T, Gandara D. Liquid Biopsy for Advanced NSCLC: A Consensus Statement From the International Association for the Study of Lung Cancer. J Thorac Oncol. 2021 Oct;16(10):1647-1662. doi: 10.1016/j.jtho.2021.06.017. Epub 2021 Jul 8. PMID: 34246791.	Review
29	Ichimura H, Nawa T, Yamamoto Y, Shimizu K, Kobayashi K, Kitazawa S, Kanbara H, Odagiri T, Endo K, Matsunaga T, Nakamura S, Yagi S, Sato Y. Detection of circulating tumor cells in patients with lung cancer using metallic micro-cavity array filter: A pilot study. Mol Clin Oncol. 2020 Mar;12(3):278-283.	Advanced stage, survival outcome not studied

	doi: 10.3892/mco.2020.1973. Epub 2020 Jan 10. PMID: 32064107; PMCID: PMC7016515.	
30	Ilie M, Long E, Butori C, Hofman V, Coelle C, Mauro V, Zahaf K, Marquette CH, Mouroux J, Paterlini-Bréchot P, Hofman P. ALK-gene rearrangement: a comparative analysis on circulating tumour cells and tumour tissue from patients with lung adenocarcinoma. Ann Oncol. 2012 Nov;23(11):2907-2913. doi: 10.1093/annonc/mds137. Epub 2012 Jun 26. PMID: 22735679.	Advanced stage, survival outcome not studied
31	Ilie M, Hofman V, Long-Mira E, Selva E, Vignaud J-M, et al. (2014) ''Sentinel'' Circulating Tumor Cells Allow Early Diagnosis of Lung Cancer in Patients with Chronic Obstructive Pulmonary Disease. PLoS ONE 9(10): e111597. doi:10.1371/journal.pone.0111597	survival outcome not studied
32	Isobe K, Hata Y, Kobayashi K, Hirota N, Sato K, Sano G, Sugino K, Sakamoto S, Takai Y, Shibuya K, Takagi K, Homma S. Clinical significance of circulating tumor cells and free DNA in non-small cell lung cancer. Anticancer Res. 2012 Aug;32(8):3339-44. PMID: 22843912.	Advanced stage, ct-DNA
33	Wei S, Guo C, He J, et al. Effect of Vein-First vs Artery-First Surgical Technique on Circulating Tumor Cells and Survival in Patients With Non–Small Cell Lung Cancer: A Randomized Clinical Trial and Registry-Based Propensity Score Matching Analysis. JAMA Surg. 2019;154(7):e190972-e190972. doi:10.1001/jamasurg.2019.0972	Advanced stage
34	Yin J, Wang Y, Yin H, Chen W, Jin G, Ma H, et al. (2015) Circulating Tumor Cells Enriched by the Depletion of Leukocytes with Bi-Antibodies in Non- Small Cell Lung Cancer: Potential Clinical Application. PLoS ONE 10(8): e0137076. doi:10.1371/journal.pone.0137076	Advanced stage
35	Kapeleris J, Kulasinghe A, Warkiani ME, Vela I, Kenny L, O'Byrne K and Punyadeera C (2018) The Prognostic Role of Circulating Tumor Cells (CTCs) in Lung Cancer. Front. Oncol. 8:311. doi: 10.3389/fonc.2018.00311	Review
36	Koh Y, Yagi S, Akamatsu H, Kanai K, Hayata A, Tokudome N, Akamatsu K, Higuchi M, Kanbara H, Nakanishi M, Ueda H, Yamamoto N. Heterogeneous Expression of Programmed Death Receptor-ligand 1 on Circulating Tumor Cells in Patients With Lung Cancer. Clin Lung Cancer. 2019 Jul;20(4):270-277.e1. doi: 10.1016/j.cllc.2019.03.004. Epub 2019 Mar 25. PMID: 31005568.	Advanced stage
37	Krebs MG, Sloane R, Priest L, et al. Evaluation and prognostic significance of circulating tumor cells in patients with non-small-cell lung cancer. J Clin Oncol Off J Am Soc Clin Oncol. 2011;29(12):1556-1563. doi:10.1200/JCO.2010.28.7045	Advanced stage
38	Krebs MG, Hou J-M, Sloane R, et al. Analysis of Circulating Tumor Cells in Patients with Non-small Cell Lung Cancer Using Epithelial Marker-Dependent and -Independent Approaches. J Thorac Oncol. 2012;7(2):306-315. doi:10.1097/JTO.0b013e31823c5c16	Advanced stage
39	1. Kurusu Y, Yamashita J, Ogawa M. Detection of circulating tumor cells by reverse transcriptase-polymerase chain reaction in patients with resectable non-small-cell lung cancer. Surgery. 1999;126(5):820-826. doi:10.1016/S0039-6060(99)70020-6	survival outcome not studied
40	Ma X-L, Xiao Z-L, Liu L, et al. Meta-analysis of circulating tumor cells as a prognostic marker in lung cancer. Asian Pac J Cancer Prev. 2012;13(4):1137-1144. doi:10.7314/apjcp.2012.13.4.1137	Meta-analysis
41	Li Z, Xu K, Tartarone A, Santarpia M, Zhu Y, Jiang G. Circulating tumor cells can predict the prognosis of patients with non-small cell lung cancer after resection: a retrospective study. Transl Lung Cancer Res Vol 10, No 2 (February 2021) Transl Lung Cancer Res. Published online 2021. https://tlcr.amegroups.com/article/view/49589	Retrospective study
42	Liu DG, Xue L, Li J, Yang Q, Peng JZ. Epithelial-mesenchymal transition and GALC expression of circulating tumor cells indicate metastasis and poor prognosis in non-small cell lung cancer. Cancer Biomark. 2018;22(3):417-426. doi: 10.3233/CBM-170995. PMID: 29758927.	Advanced stage
43	Liu L, Liao GQ, He P, Zhu H, Liu PH, Qu YM, Song XM, Xu QW, Gao Q, Zhang Y, Chen WF, Yin YH. Detection of circulating cancer cells in lung cancer patients with a panel of marker genes. Biochem Biophys Res Commun. 2008 Aug	Advanced stage, survival outcome not available,

	8;372(4):756-60. doi: 10.1016/j.bbrc.2008.05.101. Epub 2008 Jun 2. PMID: 18514066.	
44	Matsutani N, Sawabata N, Yamaguchi M, et al. Does lung cancer surgery cause circulating tumor cells?—A multicenter, prospective study. J Thorac Dis Vol 9, No 8 (August 2017) J Thorac Dis. Published online 2017. https://jtd.amegroups.com/article/view/14632	Survival outcome not studied
45	Cho M-S, Park CH, Lee S, Park HS (2020) Clinicopathological parameters for circulating tumor DNA shedding in surgically resected non- small cell lung cancer with EGFR or KRAS mutation. PLoS ONE 15(3): e0230622. https://doi. org/10.1371/journal.pone.0230622	ct-DNA
46	Murlidhar V, Reddy RM, Fouladdel S, et al. Poor Prognosis Indicated by Venous Circulating Tumor Cell Clusters in Early-Stage Lung Cancers. Cancer Res. 2017;77(18):5194-5206. doi:10.1158/0008-5472.CAN-16-2072	Use of CTC cluster
47	Nair VS, Keu KV, Luttgen MS, Kolatkar A, Vasanawala M, et al. (2013) An Observational Study of Circulating Tumor Cells and 18F-FDG PET Uptake in Patients with Treatment-Naive Non-Small Cell Lung Cancer. PLoS ONE 8(7): e67733. doi:10.1371/journal.pone.0067733	Non-interventional study
48	Navarro A, Molins L, Marrades RM, Moises J, Viñolas N, Morales S, Canals J, Castellano JJ, Ramírez J, Monzo M. Exosome Analysis in Tumor-Draining Pulmonary Vein Identifies NSCLC Patients with Higher Risk of Relapse after Curative Surgery. Cancers (Basel). 2019 Feb 21;11(2):249. doi: 10.3390/cancers11020249. PMID: 30795562; PMCID: PMC6407158.	Circulating exosome analysis
49	Ohara S, Suda K, Sakai K, Nishino M, Chiba M, Shimoji M, Takemoto T, Fujino T, Koga T, Hamada A, Soh J, Nishio K, Mitsudomi T. Prognostic implications of preoperative versus postoperative circulating tumor DNA in surgically resected lung cancer patients: a pilot study. Transl Lung Cancer Res. 2020 Oct;9(5):1915-1923. doi: 10.21037/tlcr-20-505. PMID: 33209612; PMCID: PMC7653121.	Ct-DNA
50	Okumura Y, Tanaka F, Yoneda K, et al. Circulating tumor cells in pulmonary venous blood of primary lung cancer patients. Ann Thorac Surg. 2009;87(6):1669-1675. doi:10.1016/j.athoracsur.2009.03.073	Survival outcome not studied
51	Peng H, Tan X, Wang Y, et al. Clinical significance of Ki67 and circulating tumor cells with an epithelial-mesenchymal transition phenotype in non-small cell lung cancer. Am J Transl Res. 2020;12(6):2916-2928. Published 2020 Jun 15.	Advanced stage
52	Pirozzi G, Tirino V, Camerlingo R, La Rocca A, Martucci N, Scognamiglio G, Franco R, Cantile M, Normanno N, Rocco G. Prognostic value of cancer stem cells, epithelial-mesenchymal transition and circulating tumor cells in lung cancer. Oncol Rep. 2013 May;29(5):1763-8. doi: 10.3892/or.2013.2294. Epub 2013 Feb 19. PMID: 23426441.	Non-interventional study
53	Poggiana C, Rossi E, Zamarchi R. Possible role of circulating tumor cells in early detection of lung cancer. J Thorac Dis Vol 12, No 7 (July 2020) J Thorac Dis. Published online 2020. https://jtd.amegroups.com/article/view/36574	Review
54	Punnoose EA, Atwal S, Liu W, Raja R, Fine BM, Hughes BG, Hicks RJ, Hampton GM, Amler LC, Pirzkall A, Lackner MR. Evaluation of circulating tumor cells and circulating tumor DNA in non-small cell lung cancer: association with clinical endpoints in a phase II clinical trial of pertuzumab and erlotinib. Clin Cancer Res. 2012 Apr 15;18(8):2391-401. doi: 10.1158/1078-0432.CCR-11-3148. Epub 2012 Apr 5. PMID: 22492982.	Advanced stage
55	Qi Y, Wang W. Clinical significance of circulating tumor cells in squamous cell lung cancer patients. Cancer Biomark. 2017;18(2):161-167. doi: 10.3233/CBM-160090. PMID: 27983527.	Advanced stage
56	Reddy RM, Murlidhar V, Zhao L, et al. Pulmonary venous blood sampling significantly increases the yield of circulating tumor cells in early-stage lung cancer. J Thorac Cardiovasc Surg. 2016;151(3):852-858. doi:10.1016/j.jtcvs.2015.09.126	Survival outcome not studied
57	Rolle A, Günzel R, Pachmann U, Willen B, Höffken K, Pachmann K. Increase in number of circulating disseminated epithelial cells after surgery for non-small cell lung cancer monitored by MAINTRAC(R) is a predictor for relapse: A preliminary report. World J Surg Oncol. 2005 Mar 31;3(1):18. doi: 10.1186/1477-7819-3-18. PMID: 15801980; PMCID: PMC1087511.	Non-surgical intervention

58	Jin XR, Zhu LY, Qian K, et al. Circulating tumor cells in early stage lung adenocarcinoma: a case series report and literature review. Oncotarget. 2017;8(14):23130-23141. doi:10.18632/oncotarget.15506	Case series
59	Sawabata N, Funaki S, Hyakutake T, Shintani Y, Fujiwara A, Okumura M. Perioperative circulating tumor cells in surgical patients with non-small cell lung cancer: does surgical manipulation dislodge cancer cells thus allowing them to pass into the peripheral blood? Surg Today. 2016 Dec;46(12):1402-1409. doi: 10.1007/s00595-016-1318-4. Epub 2016 Mar 7. PMID: 26951195.	Survival outcome not extractable
60	Sawabata N, Kitamura T, Nitta Y, Taketa T, Ohno T, Fukumori T, Hyakutake T, Nakamura T. Lung cancer biopsy dislodges tumor cells into circulating blood. J Cancer Metastasis Treat 2017;3:16-20. http://dx.doi.org/10.20517/2394-4722.2016.67	Survival outcome not studied
61	Sawabata N, Nakamura T, Kawaguchi T, Watanabe T, Ouji NS, Ito T, Taniguchi S. Circulating tumor cells detected only after surgery for non-small cell lung cancer: is it a predictor of recurrence? J Thorac Dis. 2020 Sep;12(9):4623-4632. doi: 10.21037/jtd-20-1636. PMID: 33145035; PMCID: PMC7578482.	Advanced stage
62	Sawabata N, Susaki Y, Nakamura T, Kawaguchi T, Yasukawa M, Taniguchi S. Cluster circulating tumor cells in surgical cases of lung cancer. Gen Thorac Cardiovasc Surg. 2020 Sep;68(9):975-983. doi: 10.1007/s11748-020-01308-3. Epub 2020 Feb 10. PMID: 32043230.	Use of CTC cluster
63	Sawabata N, Funaki S, Shintani Y, Okumura M. Lung excision of non-small-cell lung cancer leaves cancer cells in residual lobe: cytological detection using pulmonary vein blood. Interact Cardiovasc Thorac Surg. 2016 Feb;22(2):131-5. doi: 10.1093/icvts/ivv301. Epub 2015 Nov 3. PMID: 26538102.	Use of CTC cluster
64	Sawabata N, Okumura M, Utsumi T, Inoue M, Shiono H, Minami M, Nishida T, Sawa Y. Circulating tumor cells in peripheral blood caused by surgical manipulation of non-small-cell lung cancer: pilot study using an immunocytology method. Gen Thorac Cardiovasc Surg. 2007 May;55(5):189-92. doi: 10.1007/s11748-007-0101-2. PMID: 17554991.	Survival outcome not studied
65	Sher YP, Shih JY, Yang PC, Roffler SR, Chu YW, Wu CW, Yu CL, Peck K. Prognosis of non-small cell lung cancer patients by detecting circulating cancer cells in the peripheral blood with multiple marker genes. Clin Cancer Res. 2005 Jan 1;11(1):173-9. PMID: 15671543.	Advanced stage
66	Sheu CC, Yu YP, Tsai JR, Chang MY, Lin SR, Hwang JJ, Chong IW. Development of a membrane array-based multimarker assay for detection of circulating cancer cells in patients with non-small cell lung cancer. Int J Cancer. 2006 Sep 15;119(6):1419-26. doi: 10.1002/ijc.21999. PMID: 16642481.	Advanced stage
67	Song PP, Zhang W, Zhang B, Liu Q, DU J. Effects of different sequences of pulmonary artery and vein ligations during pulmonary lobectomy on blood micrometastasis of non-small cell lung cancer. Oncol Lett. 2013 Feb;5(2):463-468. doi: 10.3892/ol.2012.1022. Epub 2012 Nov 9. PMID: 23420582; PMCID: PMC3572991.	Survival outcome not studied
68	Sozzi G, Conte D, Mariani L, Lo Vullo S, Roz L, Lombardo C, Pierotti MA, Tavecchio L. Analysis of circulating tumor DNA in plasma at diagnosis and during follow-up of lung cancer patients. Cancer Res. 2001 Jun 15;61(12):4675-8. PMID: 11406535.	Ct-DNA
69	Guo, N., Lou, F., Ma, Y. et al. Circulating tumor DNA detection in lung cancer patients before and after surgery. Sci Rep 6, 33519 (2016). https://doi.org/10.1038/srep33519	Ct-DNA
70	Syrigos K, Fiste O, Charpidou A, Grapsa D. Circulating tumor cells count as a predictor of survival in lung cancer. *Crit Rev Oncol Hematol*. 2018;125:60-68. doi:https://doi.org/10.1016/j.critrevonc.2018.03.004	Review
71	Tamminga M, de Wit S, van de Wauwer C, et al. Analysis of Released Circulating Tumor Cells During Surgery for Non-Small Cell Lung Cancer. Clin Cancer Res. 2020;26(7):1656 LP - 1666. doi:10.1158/1078-0432.CCR-19-2541	Survival outcome not studied
72	Tanaka F, Yoneda K, Kondo N, Hashimoto M, Takuwa T, Matsumoto S, Okumura Y, Rahman S, Tsubota N, Tsujimura T, Kuribayashi K, Fukuoka K, Nakano T, Hasegawa S. Circulating tumor cell as a diagnostic marker in primary lung cancer.	Survival outcome not studied

	Clin Cancer Res. 2009 Nov 15;15(22):6980-6. doi: 10.1158/1078-0432.CCR-09-1095. Epub 2009 Nov 3. PMID: 19887487.	
73	Tanaka F, Yoneda K, Hasegawa S. Circulating tumor cells (CTCs) in lung cancer: current status and future perspectives. Lung Cancer (Auckl). 2010 Jul 3;1:77-84. doi: 10.2147/lctt.s6828. PMID: 28210108; PMCID: PMC5312474.	Review
74	Tarumi, S., Gotoh, M., Kasai, Y. et al. Innovative method using circulating tumor cells for prediction of the effects of induction therapy on locally advanced non-small cell lung cancer. J Cardiothorac Surg 8, 175 (2013). https://doi.org/10.1186/1749-8090-8-175	Non-surgical intervention
75	Tognela A, Spring KJ, Becker T, et al. Predictive and prognostic value of circulating tumor cell detection in lung cancer: a clinician's perspective. Crit Rev Oncol Hematol. 2015;93(2):90-102. doi:10.1016/j.critrevonc.2014.10.001	Review
76	1. Togo S, Katagiri N, Namba Y, et al. Sensitive detection of viable circulating tumor cells using a novel conditionally telomerase-selective replicating adenovirus in non-small cell lung cancer patients. Oncotarget. 2017;8(21):34884-34895. doi:10.18632/oncotarget.16818	Advanced stage

Table S1. PRISMA Abstract Checklist

Section and Topic	Item #	Checklist item	Reported (Yes/No)
**TITLE**			
Title	1	Identify the report as a systematic review.	Yes
**BACKGROUND**			
Objectives	2	Provide an explicit statement of the main objective(s) or question(s) the review addresses.	Yes
**METHODS**			
Eligibility criteria	3	Specify the inclusion and exclusion criteria for the review.	Yes
Information sources	4	Specify the information sources (e.g. databases, registers) used to identify studies and the date when each was last searched.	Yes
Risk of bias	5	Specify the methods used to assess risk of bias in the included studies.	Yes
Synthesis of results	6	Specify the methods used to present and synthesise results.	Yes
**RESULTS**			
Included studies	7	Give the total number of included studies and participants and summarise relevant characteristics of studies.	Yes
Synthesis of results	8	Present results for main outcomes, preferably indicating the number of included studies and participants for each. If meta-analysis was done, report the summary estimate and confidence/credible interval. If comparing groups, indicate the direction of the effect (i.e. which group is favoured).	Yes
**DISCUSSION**			
Limitations of evidence	9	Provide a brief summary of the limitations of the evidence included in the review (e.g. study risk of bias, inconsistency and imprecision).	No
Interpretation	10	Provide a general interpretation of the results and important implications.	Yes
**OTHER**			
Funding	11	Specify the primary source of funding for the review.	Yes
Registration	12	Provide the register name and registration number.	No

From: Page MJ, McKenzie JE, Bossuyt PM, Boutron I, Hoffmann TC, Mulrow CD, et al. The PRISMA 2020 statement: an updated guideline for reporting systematic reviews. BMJ 2021;372:n71. doi: 10.1136/bmj.n71

Table S2. PRISMA 2020 Chceklist

Section and Topic	Item #	Checklist item	Location where item is reported
**TITLE**			
Title	1	Identify the report as a systematic review.	Title
**ABSTRACT**			
Abstract	2	See the PRISMA 2020 for Abstracts checklist.	Abstract
**INTRODUCTION**			
Rationale	3	Describe the rationale for the review in the context of existing knowledge.	Introduction
Objectives	4	Provide an explicit statement of the objective(s) or question(s) the review addresses.	Introduction
**METHODS**			
Eligibility criteria	5	Specify the inclusion and exclusion criteria for the review and how studies were grouped for the syntheses.	Methods, Study selection
Information sources	6	Specify all databases, registers, websites, organisations, reference lists and other sources searched or consulted to identify studies. Specify the date when each source was last searched or consulted.	Methods, Data sources
Search strategy	7	Present the full search strategies for all databases, registers and websites, including any filters and limits used.	Methods, Search strategy
Selection process	8	Specify the methods used to decide whether a study met the inclusion criteria of the review, including how many reviewers screened each record and each report retrieved, whether they worked independently, and if applicable, details of automation tools used in the process.	Methods, Data abstraction
Data collection process	9	Specify the methods used to collect data from reports, including how many reviewers collected data from each report, whether they worked independently, any processes for obtaining or confirming data from study investigators, and if applicable, details of automation tools used in the process.	Methods, Data abstraction
Data items	10a	List and define all outcomes for which data were sought. Specify whether all results that were compatible with each outcome domain in each study were sought (e.g. for all measures, time points, analyses), and if not, the methods used to decide which results to collect.	Methods, Data abstraction
	10b	List and define all other variables for which data were sought (e.g. participant and intervention characteristics, funding sources). Describe any assumptions made about any missing or unclear information.	Methods, Data abstraction
Study risk of bias assessment	11	Specify the methods used to assess risk of bias in the included studies, including details of the tool(s) used, how many reviewers assessed each study and whether they worked independently, and if applicable, details of automation tools used in the process.	Methods, Data abstraction

Section and Topic	Item #	Checklist item	Location where item is reported
Effect measures	12	Specify for each outcome the effect measure(s) (e.g. risk ratio, mean difference) used in the synthesis or presentation of results.	Methods, Data abstraction
Synthesis methods	13a	Describe the processes used to decide which studies were eligible for each synthesis (e.g. tabulating the study intervention characteristics and comparing against the planned groups for each synthesis (item #5)).	Statistical Analysis
	13b	Describe any methods required to prepare the data for presentation or synthesis, such as handling of missing summary statistics, or data conversions.	Statistical Analysis
	13c	Describe any methods used to tabulate or visually display results of individual studies and syntheses.	Statistical Analysis
	13d	Describe any methods used to synthesize results and provide a rationale for the choice(s). If meta-analysis was performed, describe the model(s), method(s) to identify the presence and extent of statistical heterogeneity, and software package(s) used.	Statistical Analysis
	13e	Describe any methods used to explore possible causes of heterogeneity among study results (e.g. subgroup analysis, meta-regression).	Statistical Analysis
	13f	Describe any sensitivity analyses conducted to assess robustness of the synthesized results.	Statistical Analysis
Reporting bias assessment	14	Describe any methods used to assess risk of bias due to missing results in a synthesis (arising from reporting biases).	Statistical Analysis
Certainty assessment	15	Describe any methods used to assess certainty (or confidence) in the body of evidence for an outcome.	-
**RESULTS**			
Study selection	16a	Describe the results of the search and selection process, from the number of records identified in the search to the number of studies included in the review, ideally using a flow diagram.	Results
	16b	Cite studies that might appear to meet the inclusion criteria, but which were excluded, and explain why they were excluded.	Supplemental file
Study characteristics	17	Cite each included study and present its characteristics.	Study characteristics
Risk of bias in studies	18	Present assessments of risk of bias for each included study.	Quality of included studies
Results of individual studies	19	For all outcomes, present, for each study: (a) summary statistics for each group (where appropriate) and (b) an effect estimate and its precision (e.g. confidence/credible interval), ideally using structured tables or plots.	Meta-analysis
Results of syntheses	20a	For each synthesis, briefly summarise the characteristics and risk of bias among contributing studies.	Meta-analysis
	20b	Present results of all statistical syntheses conducted. If meta-analysis was done, present for each the summary estimate and its precision (e.g.	Meta-analysis

Section and Topic	Item #	Checklist item	Location where item is reported
		confidence/credible interval) and measures of statistical heterogeneity. If comparing groups, describe the direction of the effect.	
	20c	Present results of all investigations of possible causes of heterogeneity among study results.	Meta-analysis
	20d	Present results of all sensitivity analyses conducted to assess the robustness of the synthesized results.	Meta-analysis
Reporting biases	21	Present assessments of risk of bias due to missing results (arising from reporting biases) for each synthesis assessed.	-
Certainty of evidence	22	Present assessments of certainty (or confidence) in the body of evidence for each outcome assessed.	-
**DISCUSSION**			
Discussion	23a	Provide a general interpretation of the results in the context of other evidence.	Discussion
	23b	Discuss any limitations of the evidence included in the review.	Discussion
	23c	Discuss any limitations of the review processes used.	Discussion
	23d	Discuss implications of the results for practice, policy, and future research.	Discussion
**OTHER INFORMATION**			
Registration and protocol	24a	Provide registration information for the review, including register name and registration number, or state that the review was not registered.	Not registered
	24b	Indicate where the review protocol can be accessed, or state that a protocol was not prepared.	Not prepared
	24c	Describe and explain any amendments to information provided at registration or in the protocol.	None
Support	25	Describe sources of financial or non-financial support for the review, and the role of the funders or sponsors in the review.	Post manuscript notes
Competing interests	26	Declare any competing interests of review authors.	No competing interest
Availability of data, code and other materials	27	Report which of the following are publicly available and where they can be found: template data collection forms; data extracted from included studies; data used for all analyses; analytic code; any other materials used in the review.	Data extracted from included studies

From: Page MJ, McKenzie JE, Bossuyt PM, Boutron I, Hoffmann TC, Mulrow CD, et al. The PRISMA 2020 statement: an updated guideline for reporting systematic reviews. BMJ 2021;372:n71. doi: 10.1136/bmj.n71

For more information, visit: http://www.prisma-statement.org/

Table S3. Study quality score

References	Selection				Comparability		Outcome			Score
	REC	SNEC	AE	DO	SC	AF	AO	FU	FUO	
Bayarri-Lara 2016, Spain	★	★	★	★	★		★	★	★	8
Chemi 2019, UK	★	★	★	★	★	★	★	★	★	9
Chudasama 2017, UK	★		★	★	★		★			5
Crosbie 2016, UK	★	★	★	★	★	★	★		★	8
Dandachi 2017, Austria	★	★		★	★	★			★	6
Dong 2019, China	★	★	★	★		★	★		★	7
Funaki 2012, Japan	★		★						★	3
Hashimito 2017, Japan	★	★		★	★	★	★	★		7
Hofman 2010,France	★			★	★	★	★	★		6
Li Jian 2014, China	★	★	★		★	★		★	★	7
Li Yunsong 2017, China	★	★		★	★	★		★		6
Li hang 2021, China	★	★	★	★		★	★		★	7
Manjunath 2019, USA	★	★		★	★	★	★	★		7
Miguel-Perez 2019, Spain	★	★	★		★	★	★	★	★	8
Sienel 2003, Germany	★		★				★			3
Yamashita 2002, Japan	★		★	★			★	★		5
Yoon 2011, South Korea	★	★	★	★	★			★	★	7
Zhu 2013, China	★	★	★	★	★	★	★	★		8

REC: representativeness of the exposed cohort; SNEC: selection of the non-exposed cohort; AE: ascertainment of exposure; DO: demonstration that outcome of interest was not present at the start of study; SC: study controls for age, sex; AF: study controls for any additional factors; AO: assessment of outcome; FU: follow-up long enough for outcomes to occur; FUO: adequacy of follow-up of cohorts.

Figure S1. Sensitivity analysis: Correlation of CTC with Disease-free survival (Chemi et al. excluded)

Study or Subgroup	log[Hazard Ratio]	SE	Weight	Hazard Ratio IV, Random, 95% CI
**6.2.1 Baseline CTC**				
Bayarri-Lara 2016	0.29266961	0.52168394	4.6%	1.34 [0.48, 3.73]
Chemi 2019	0.15700375	0.06954925	0.0%	1.17 [1.02, 1.34]
Crosbie 2016	1.66013103	0.64678861	3.1%	5.26 [1.48, 18.69]
Dandachi 2017	1.70583841	0.61791052	3.4%	5.51 [1.64, 18.48]
Dong 2019	1.56064775	0.32287407	10.1%	4.76 [2.53, 8.97]
Funaki 2012	1.93516805	0.74387743	2.4%	6.93 [1.61, 29.76]
Hang Li 2021	1.41047465	0.61356628	3.4%	4.10 [1.23, 13.64]
Hashimoto 2017	1.08530127	0.60863511	3.5%	2.96 [0.90, 9.76]
Hofman 2010	0.967364	0.27916361	12.4%	2.63 [1.52, 4.55]
Jian Li 2014	0.29266961	0.35068592	8.9%	1.34 [0.67, 2.66]
Manjunath 2019	1.09901237	1.11863425	1.1%	3.00 [0.34, 26.88]
Yoon 2011	2.00417906	1.04403238	1.2%	7.42 [0.96, 57.42]
Yunsong Li 2017	2.16447179	0.80259428	2.1%	8.71 [1.81, 41.99]
Zhu 2013	0.92703283	0.33769022	9.4%	2.53 [1.30, 4.90]
**Subtotal (95% CI)**			**65.5%**	**3.09 [2.25, 4.25]**
Heterogeneity: $Tau^2 = 0.08$; $Chi^2 = 15.95$, df = 12 (P = 0.19); $I^2 = 25\%$				
Test for overall effect: Z = 6.98 (P < 0.00001)				
**6.2.2 Postoperative CTC**				
Bayarri-Lara 2016	1.74919985	0.68451582	2.8%	5.75 [1.50, 22.00]
Jian Li 2014	0.76080583	0.26602575	13.3%	2.14 [1.27, 3.60]
Miguel-Perez 2019	0.92028275	0.43423367	6.3%	2.51 [1.07, 5.88]
Yoon 2011	0.13102826	1.43614145	0.7%	1.14 [0.07, 19.03]
Yunsong Li 2017	2.55567572	1.06092375	1.2%	12.88 [1.61, 103.03]
Zhu 2013	1.14485954	0.31735821	10.3%	3.14 [1.69, 5.85]
**Subtotal (95% CI)**			**34.5%**	**2.73 [1.94, 3.85]**
Heterogeneity: $Tau^2 = 0.00$; $Chi^2 = 4.76$, df = 5 (P = 0.45); $I^2 = 0\%$				
Test for overall effect: Z = 5.77 (P < 0.00001)				
**Total (95% CI)**			**100.0%**	**2.95 [2.34, 3.73]**
Heterogeneity: $Tau^2 = 0.03$; $Chi^2 = 20.86$, df = 18 (P = 0.29); $I^2 = 14\%$				
Test for overall effect: Z = 9.16 (P < 0.00001)				
Test for subgroup differences: $Chi^2 = 0.27$, df = 1 (P = 0.60), $I^2 = 0\%$				

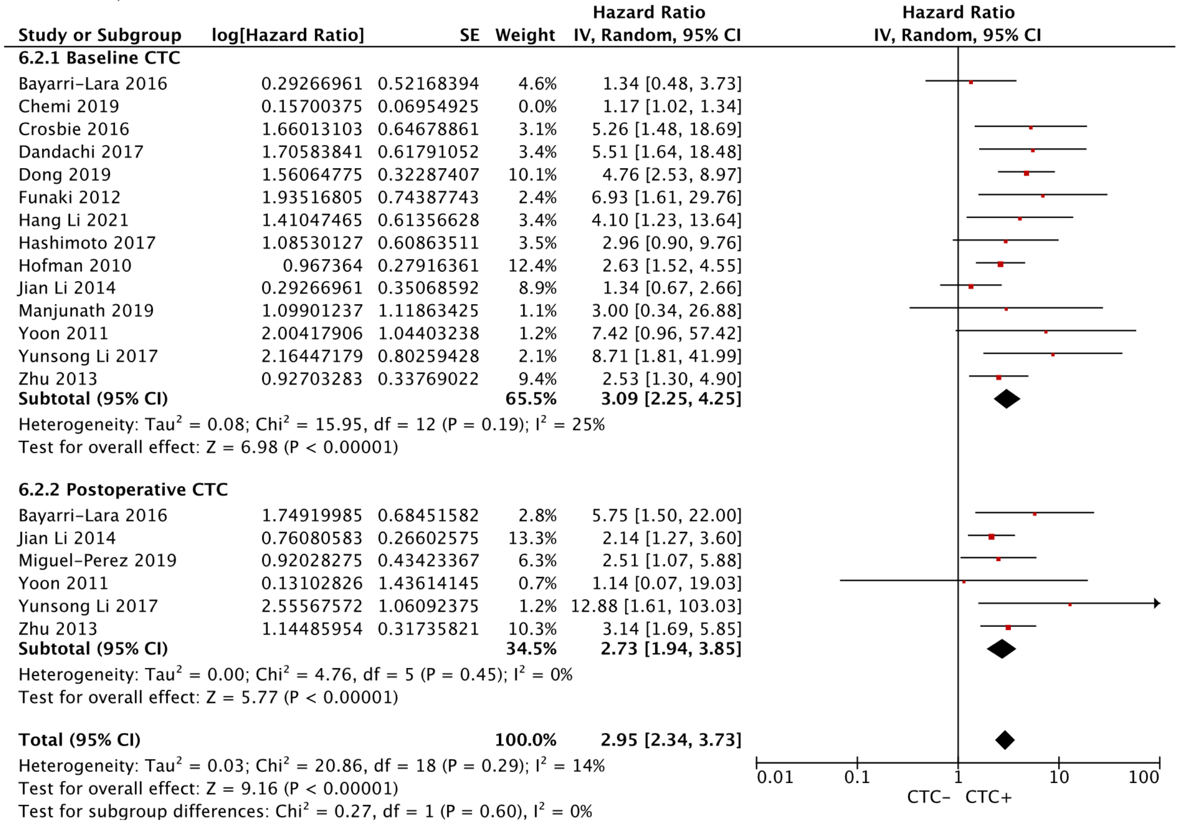

Figure S2. Subgroup analysis: Source of blood collection

A. Overall Survival

Study or Subgroup	log[Hazard Ratio]	SE	Weight	Hazard Ratio IV, Random, 99% CI
**7.1.1 Peripheral Source**				
Chudasama 2017	3.11159216	0.9345738	3.9%	22.46 [2.02, 249.36]
Crosbie 2016	1.31640823	0.59236033	7.4%	3.73 [0.81, 17.15]
Dong 2019	1.27296568	0.48279698	9.2%	3.57 [1.03, 12.39]
Hang Li 2021	0.90138012	0.62708218	6.9%	2.46 [0.49, 12.39]
Hofman 2010	0.74003077	0.23163059	14.6%	2.10 [1.15, 3.81]
Jian Li 2014	0.3435897	0.34133865	12.1%	1.41 [0.59, 3.40]
Yunsong Li 2017	2.12942147	0.7895975	5.1%	8.41 [1.10, 64.28]
**Subtotal (95% CI)**			**59.2%**	**2.99 [1.78, 5.03]**
Heterogeneity: $Tau^2 = 0.22$; $Chi^2 = 12.24$, df = 6 (P = 0.06); $I^2 = 51\%$				
Test for overall effect: Z = 4.14 (P < 0.0001)				
**7.1.2 Pulmonary Vein**				
Crosbie 2016	0.28517894	0.11595784	16.8%	1.33 [0.99, 1.79]
Dong 2019	1.27296568	0.48279698	9.2%	3.57 [1.03, 12.39]
Hashimoto 2017	1.4545732	0.70952722	5.9%	4.28 [0.69, 26.63]
Sienel 2003	1.43508453	0.49411772	9.0%	4.20 [1.18, 15.00]
**Subtotal (95% CI)**			**40.8%**	**2.68 [1.24, 5.77]**
Heterogeneity: $Tau^2 = 0.41$; $Chi^2 = 10.82$, df = 3 (P = 0.01); $I^2 = 72\%$				
Test for overall effect: Z = 2.52 (P = 0.01)				
**Total (95% CI)**			**100.0%**	**2.82 [1.86, 4.26]**
Heterogeneity: $Tau^2 = 0.25$; $Chi^2 = 29.03$, df = 10 (P = 0.001); $I^2 = 66\%$				
Test for overall effect: Z = 4.92 (P < 0.00001)				
Test for subgroup differences: $Chi^2 = 0.06$, df = 1 (P = 0.81), $I^2 = 0\%$				

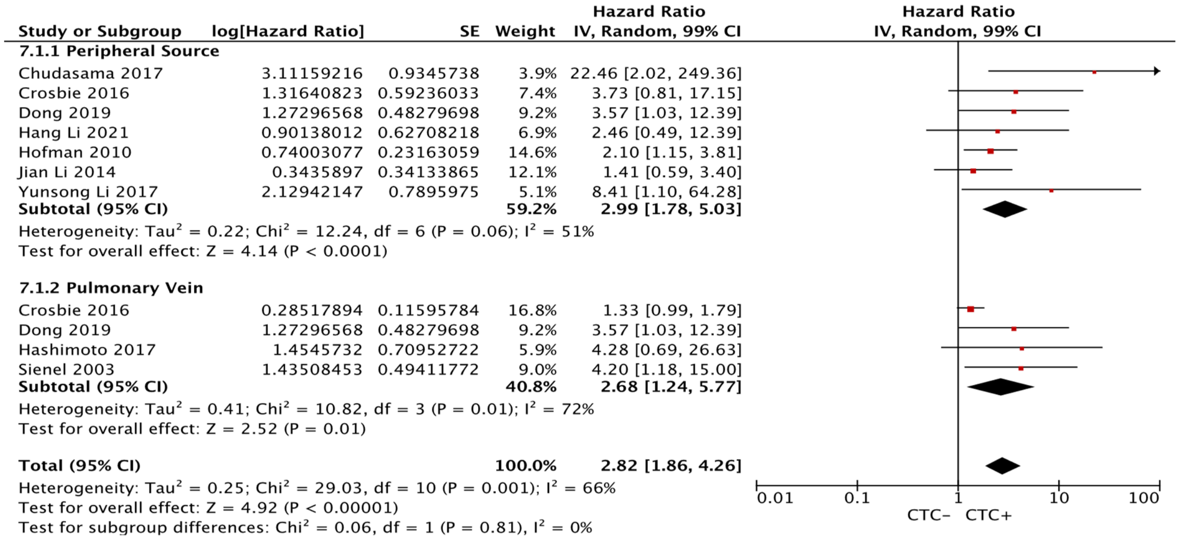

B. Disease-Free Survival

Study or Subgroup	log[Hazard Ratio]	SE	Weight	Hazard Ratio IV, Random, 99% CI
**7.2.1 Peripheral Source**				
Bayarri-Lara 2016	0.29266961	0.52168394	6.3%	1.34 [0.35, 5.14]
Crosbie 2016	1.66013103	0.64678861	4.9%	5.26 [0.99, 27.83]
Dandachi 2017	1.70583841	0.61791052	5.1%	5.51 [1.12, 27.04]
Hang Li 2021	1.41047465	0.61356628	5.2%	4.10 [0.84, 19.90]
Hofman 2010	0.967364	0.27916361	10.1%	2.63 [1.28, 5.40]
Jian Li 2014	0.29266961	0.35068592	8.9%	1.34 [0.54, 3.31]
Yoon 2011	2.00417906	1.04403238	2.4%	7.42 [0.50, 109.23]
Yunsong Li 2017	2.16447179	0.80259428	3.6%	8.71 [1.10, 68.84]
Zhu 2013	0.92703283	0.33769022	9.1%	2.53 [1.06, 6.03]
**Subtotal (95% CI)**			**55.5%**	**2.77 [1.88, 4.08]**
Heterogeneity: Tau^2 = 0.10; Chi^2 = 11.68, df = 8 (P = 0.17); I^2 = 32%				
Test for overall effect: Z = 5.13 (P < 0.00001)				
**7.2.2 Pulmonary vein**				
Chemi 2019	0.15700375	0.06954925	13.2%	1.17 [0.98, 1.40]
Crosbie 2016	0.41210965	0.12900108	12.6%	1.51 [1.08, 2.11]
Dong 2019	1.56064775	0.32287407	9.4%	4.76 [2.07, 10.94]
Funaki 2012	1.93516805	0.74387743	4.0%	6.93 [1.02, 47.05]
Hashimoto 2017	1.08530127	0.60863511	5.2%	2.96 [0.62, 14.20]
**Subtotal (95% CI)**			**44.5%**	**2.16 [1.33, 3.52]**
Heterogeneity: Tau^2 = 0.20; Chi^2 = 26.61, df = 4 (P < 0.0001); I^2 = 85%				
Test for overall effect: Z = 3.09 (P = 0.002)				
**Total (95% CI)**			**100.0%**	**2.58 [1.82, 3.66]**
Heterogeneity: Tau^2 = 0.23; Chi^2 = 55.24, df = 13 (P < 0.00001); I^2 = 76%				
Test for overall effect: Z = 5.32 (P < 0.00001)				
Test for subgroup differences: Chi^2 = 0.60, df = 1 (P = 0.44), I^2 = 0%				

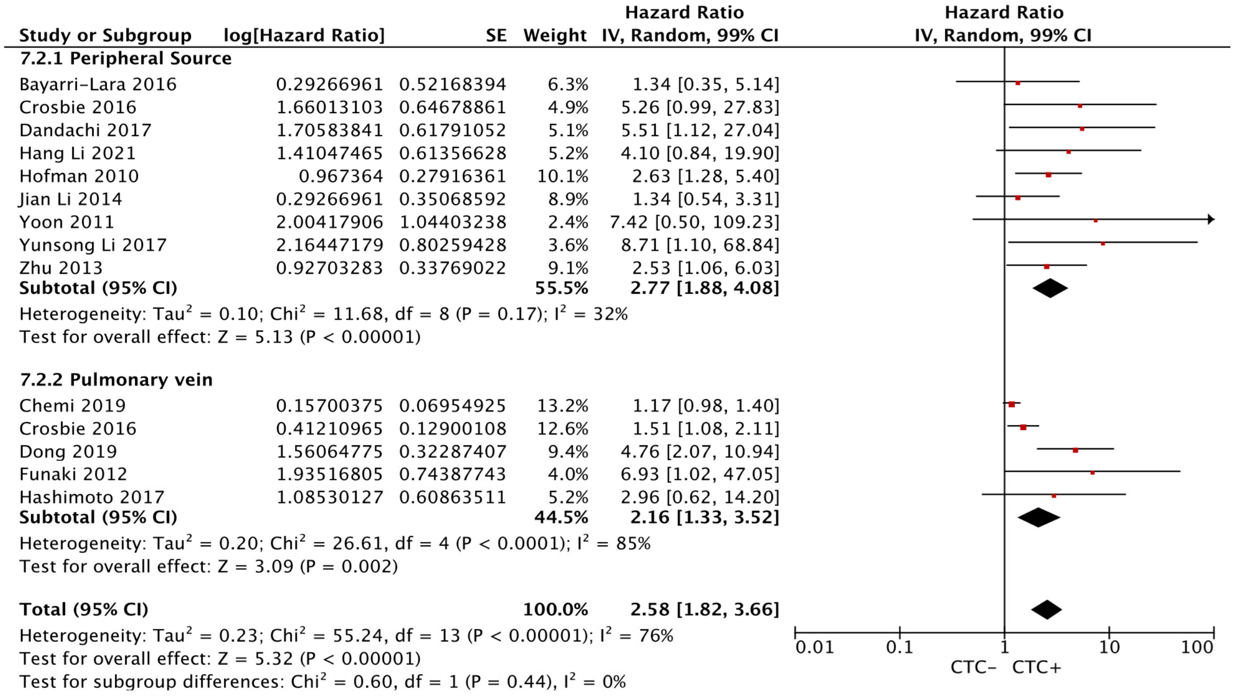

Figure S3. Subgroup analysis: Detection methods

A. Overall Survival

Study or Subgroup	log[Hazard Ratio]	SE	Weight	Hazard Ratio IV, Random, 99% CI
**8.1.1 Label Dependent**				
Crosbie 2016	1.31640823	0.59236033	8.5%	3.73 [0.81, 17.15]
Hang Li 2021	0.90138012	0.62708218	7.9%	2.46 [0.49, 12.39]
Hashimoto 2017	1.4545732	0.70952722	6.8%	4.28 [0.69, 26.63]
Yunsong Li 2017	2.12942147	0.7895975	5.9%	8.41 [1.10, 64.28]
**Subtotal (95% CI)**			**29.0%**	**3.95 [2.06, 7.60]**
Heterogeneity: Tau^2 = 0.00; Chi^2 = 1.51, df = 3 (P = 0.68); I^2 = 0%				
Test for overall effect: Z = 4.12 (P < 0.0001)				
**8.1.2 Label free**				
Chudasama 2017	3.11159216	0.9345738	4.6%	22.46 [2.02, 249.36]
Dong 2019	1.27296568	0.48279698	10.4%	3.57 [1.03, 12.39]
Hofman 2010	0.74003077	0.23163059	16.3%	2.10 [1.15, 3.81]
Manjunath 2019	1.98267787	1.05486012	3.8%	7.26 [0.48, 109.93]
Sienel 2003	1.43508453	0.49411772	10.2%	4.20 [1.18, 15.00]
**Subtotal (95% CI)**			**45.3%**	**3.91 [2.01, 7.62]**
Heterogeneity: Tau^2 = 0.27; Chi^2 = 8.26, df = 4 (P = 0.08); I^2 = 52%				
Test for overall effect: Z = 4.01 (P < 0.0001)				
**8.1.3 RTPCR**				
Jian Li 2014	0.3435897	0.34133865	13.6%	1.41 [0.59, 3.40]
Yamashita 2002	2.10619628	0.40785153	12.0%	8.22 [2.87, 23.49]
**Subtotal (95% CI)**			**25.7%**	**3.36 [0.60, 18.88]**
Heterogeneity: Tau^2 = 1.41; Chi^2 = 10.98, df = 1 (P = 0.0009); I^2 = 91%				
Test for overall effect: Z = 1.37 (P = 0.17)				
**Total (95% CI)**			**100.0%**	**3.76 [2.40, 5.89]**
Heterogeneity: Tau^2 = 0.27; Chi^2 = 21.51, df = 10 (P = 0.02); I^2 = 54%				
Test for overall effect: Z = 5.79 (P < 0.00001)				
Test for subgroup differences: Chi^2 = 0.03, df = 2 (P = 0.98), I^2 = 0%				

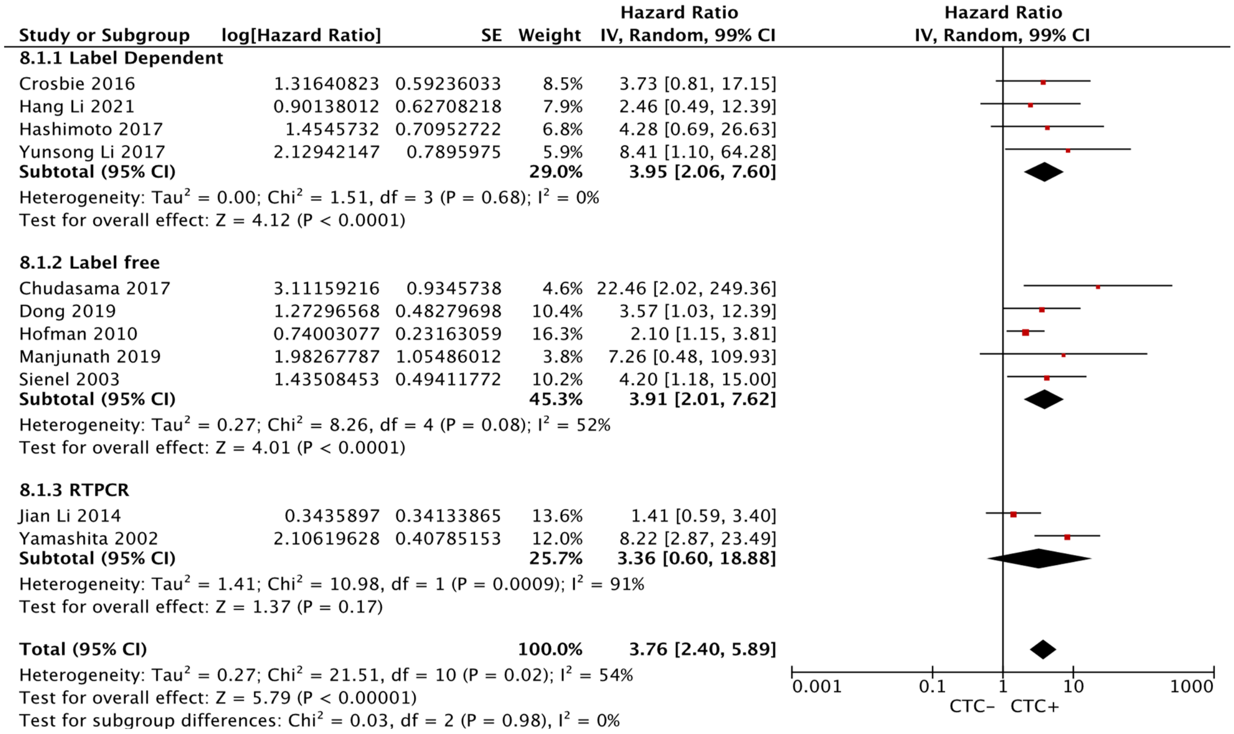

B. Disease-Free Survival

Study or Subgroup	log[Hazard Ratio]	SE	Weight	Hazard Ratio IV, Random, 99% CI
**8.2.1 Label dependent**				
Bayarri-Lara 2016	0.29266961	0.52168394	7.4%	1.34 [0.35, 5.14]
Chemi 2019	0.15700375	0.06954925	12.0%	1.17 [0.98, 1.40]
Crosbie 2016	1.66013103	0.64678861	6.1%	5.26 [0.99, 27.83]
Hang Li 2021	1.41047465	0.61356628	6.4%	4.10 [0.84, 19.90]
Hashimoto 2017	1.08530127	0.60863511	6.4%	2.96 [0.62, 14.20]
Yunsong Li 2017	2.16447179	0.80259428	4.8%	8.71 [1.10, 68.84]
**Subtotal (95% CI)**			**43.1%**	**2.62 [1.28, 5.34]**
Heterogeneity: $Tau^2 = 0.50$; $Chi^2 = 17.45$, df = 5 (P = 0.004); $I^2 = 71\%$				
Test for overall effect: Z = 2.64 (P = 0.008)				
**8.2.2 Label free**				
Dandachi 2017	1.70583841	0.61791052	6.3%	5.51 [1.12, 27.04]
Dong 2019	1.56064775	0.32287407	9.7%	4.76 [2.07, 10.94]
Funaki 2012	1.93516805	0.74387743	5.2%	6.93 [1.02, 47.05]
Hofman 2010	0.967364	0.27916361	10.3%	2.63 [1.28, 5.40]
Manjunath 2019	1.09901237	1.11863425	3.0%	3.00 [0.17, 53.54]
**Subtotal (95% CI)**			**34.6%**	**3.71 [2.55, 5.38]**
Heterogeneity: $Tau^2 = 0.00$; $Chi^2 = 3.26$, df = 4 (P = 0.52); $I^2 = 0\%$				
Test for overall effect: Z = 6.89 (P < 0.00001)				
**8.2.3 RTPCR**				
Jian Li 2014	0.29266961	0.35068592	9.4%	1.34 [0.54, 3.31]
Yoon 2011	2.00417906	1.04403238	3.4%	7.42 [0.50, 109.23]
Zhu 2013	0.92703283	0.33769022	9.6%	2.53 [1.06, 6.03]
**Subtotal (95% CI)**			**22.3%**	**2.11 [1.08, 4.12]**
Heterogeneity: $Tau^2 = 0.14$; $Chi^2 = 3.36$, df = 2 (P = 0.19); $I^2 = 40\%$				
Test for overall effect: Z = 2.19 (P = 0.03)				
**Total (95% CI)**			**100.0%**	**2.95 [1.90, 4.59]**
Heterogeneity: $Tau^2 = 0.42$; $Chi^2 = 55.54$, df = 13 (P < 0.00001); $I^2 = 77\%$				
Test for overall effect: Z = 4.81 (P < 0.00001)				
Test for subgroup differences: $Chi^2 = 2.36$, df = 2 (P = 0.31), $I^2 = 15.3\%$				

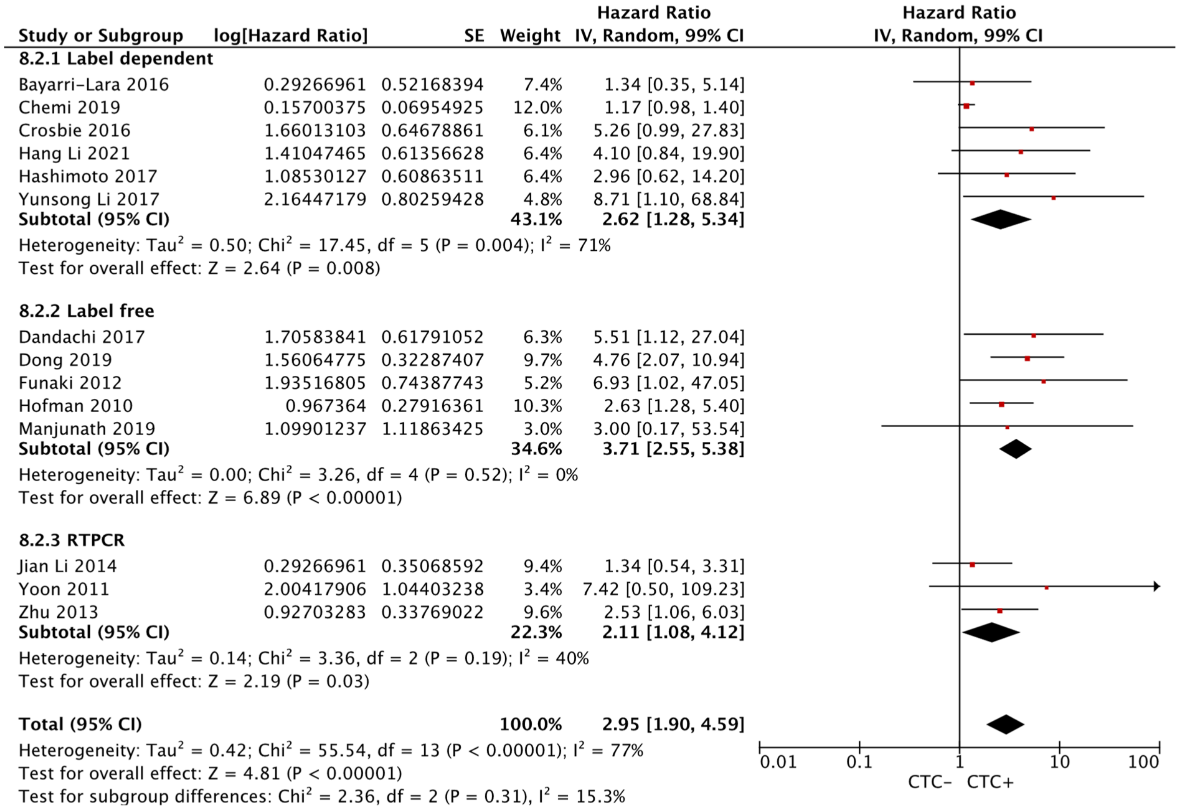

Figure S4. Subgroup analysis: Duration of follow-up

A. Overall Survival

Study or Subgroup	log[Hazard Ratio]	SE	Weight	Hazard Ratio IV, Fixed, 99% CI
**9.1.1 > 24 months**				
Chudasama 2017	3.11159216	0.9345738	2.2%	22.46 [2.02, 249.36]
Dong 2019	1.27296568	0.48279698	8.1%	3.57 [1.03, 12.39]
Hang Li 2021	0.90138012	0.62708218	4.8%	2.46 [0.49, 12.39]
Hashimoto 2017	1.4545732	0.70952722	3.8%	4.28 [0.69, 26.63]
Jian Li 2014	0.3435897	0.34133865	16.3%	1.41 [0.59, 3.40]
Sienel 2003	1.43508453	0.49411772	7.8%	4.20 [1.18, 15.00]
Yamashita 2002	2.10619628	0.40785153	11.4%	8.22 [2.87, 23.49]
Yunsong Li 2017	2.12942147	0.7895975	3.0%	8.41 [1.10, 64.28]
**Subtotal (95% CI)**			**57.5%**	**3.64 [2.55, 5.20]**
Heterogeneity: $Chi^2 = 17.15$, df = 7 (P = 0.02); $I^2 = 59\%$				
Test for overall effect: Z = 7.11 (P < 0.00001)				
**9.1.2 < 24 months**				
Crosbie 2016	1.31640823	0.59236033	5.4%	3.73 [0.81, 17.15]
Hofman 2010	0.74003077	0.23163059	35.4%	2.10 [1.15, 3.81]
Manjunath 2019	1.98267787	1.05486012	1.7%	7.26 [0.48, 109.93]
**Subtotal (95% CI)**			**42.5%**	**2.37 [1.57, 3.59]**
Heterogeneity: $Chi^2 = 1.99$, df = 2 (P = 0.37); $I^2 = 0\%$				
Test for overall effect: Z = 4.08 (P < 0.0001)				
**Total (95% CI)**			**100.0%**	**3.03 [2.32, 3.98]**
Heterogeneity: $Chi^2 = 21.51$, df = 10 (P = 0.02); $I^2 = 54\%$				
Test for overall effect: Z = 8.05 (P < 0.00001)				
Test for subgroup differences: $Chi^2 = 2.37$, df = 1 (P = 0.12), $I^2 = 57.8\%$				

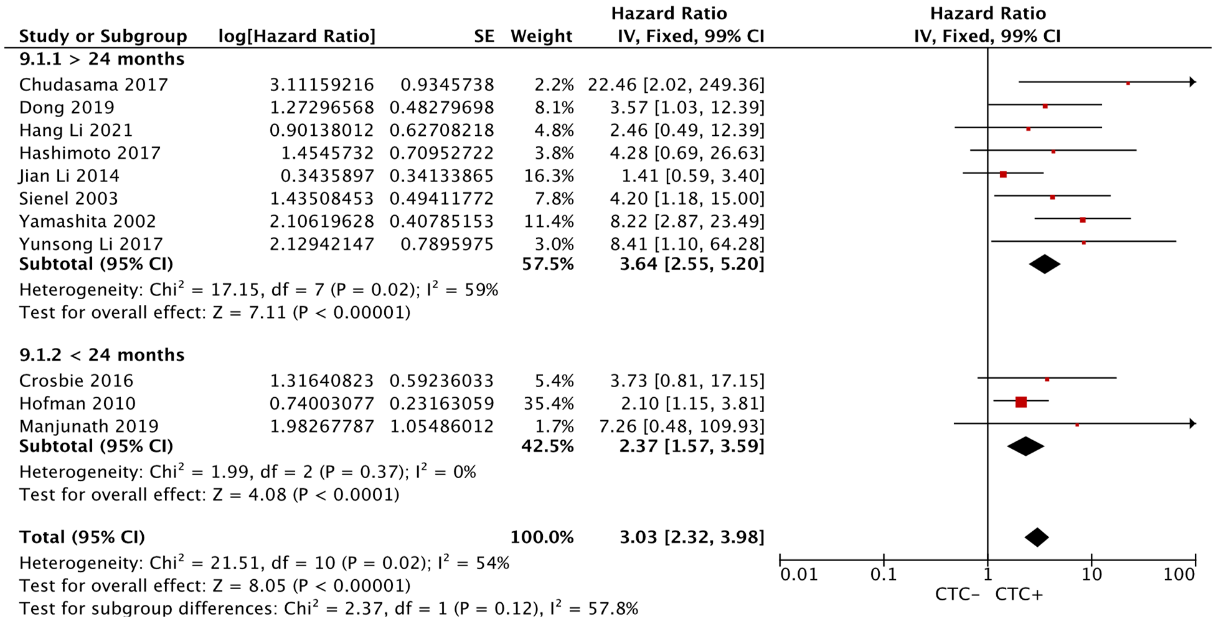

B. Disease-free Survival:

Study or Subgroup	log[Hazard Ratio]	SE	Weight	Hazard Ratio IV, Random, 99% CI
**9.2.1 >24 months**				
Chemi 2019	0.15700375	0.06954925	12.0%	1.17 [0.98, 1.40]
Dong 2019	1.56064775	0.32287407	9.7%	4.76 [2.07, 10.94]
Hang Li 2021	1.41047465	0.61356628	6.4%	4.10 [0.84, 19.90]
Hashimoto 2017	1.08530127	0.60863511	6.4%	2.96 [0.62, 14.20]
Jian Li 2014	0.29266961	0.35068592	9.4%	1.34 [0.54, 3.31]
Yoon 2011	2.00417906	1.04403238	3.4%	7.42 [0.50, 109.23]
Yunsong Li 2017	2.16447179	0.80259428	4.8%	8.71 [1.10, 68.84]
Zhu 2013	0.92703283	0.33769022	9.6%	2.53 [1.06, 6.03]
**Subtotal (95% CI)**			**61.7%**	**2.75 [1.54, 4.93]**
Heterogeneity: $Tau^2 = 0.46$; $Chi^2 = 35.90$, df = 7 (P < 0.00001); $I^2 = 80\%$				
Test for overall effect: Z = 3.40 (P = 0.0007)				
**9.2.2 < 24 months**				
Bayarri-Lara 2016	0.29266961	0.52168394	7.4%	1.34 [0.35, 5.14]
Crosbie 2016	1.66013103	0.64678861	6.1%	5.26 [0.99, 27.83]
Dandachi 2017	1.70583841	0.61791052	6.3%	5.51 [1.12, 27.04]
Funaki 2012	1.93516805	0.74387743	5.2%	6.93 [1.02, 47.05]
Hofman 2010	0.967364	0.27916361	10.3%	2.63 [1.28, 5.40]
Manjunath 2019	1.09901237	1.11863425	3.0%	3.00 [0.17, 53.54]
**Subtotal (95% CI)**			**38.3%**	**3.06 [1.95, 4.79]**
Heterogeneity: $Tau^2 = 0.04$; $Chi^2 = 5.59$, df = 5 (P = 0.35); $I^2 = 11\%$				
Test for overall effect: Z = 4.87 (P < 0.00001)				
**Total (95% CI)**			**100.0%**	**2.95 [1.90, 4.59]**
Heterogeneity: $Tau^2 = 0.42$; $Chi^2 = 55.54$, df = 13 (P < 0.00001); $I^2 = 77\%$				
Test for overall effect: Z = 4.81 (P < 0.00001)				
Test for subgroup differences: $Chi^2 = 0.08$, df = 1 (P = 0.78), $I^2 = 0\%$				

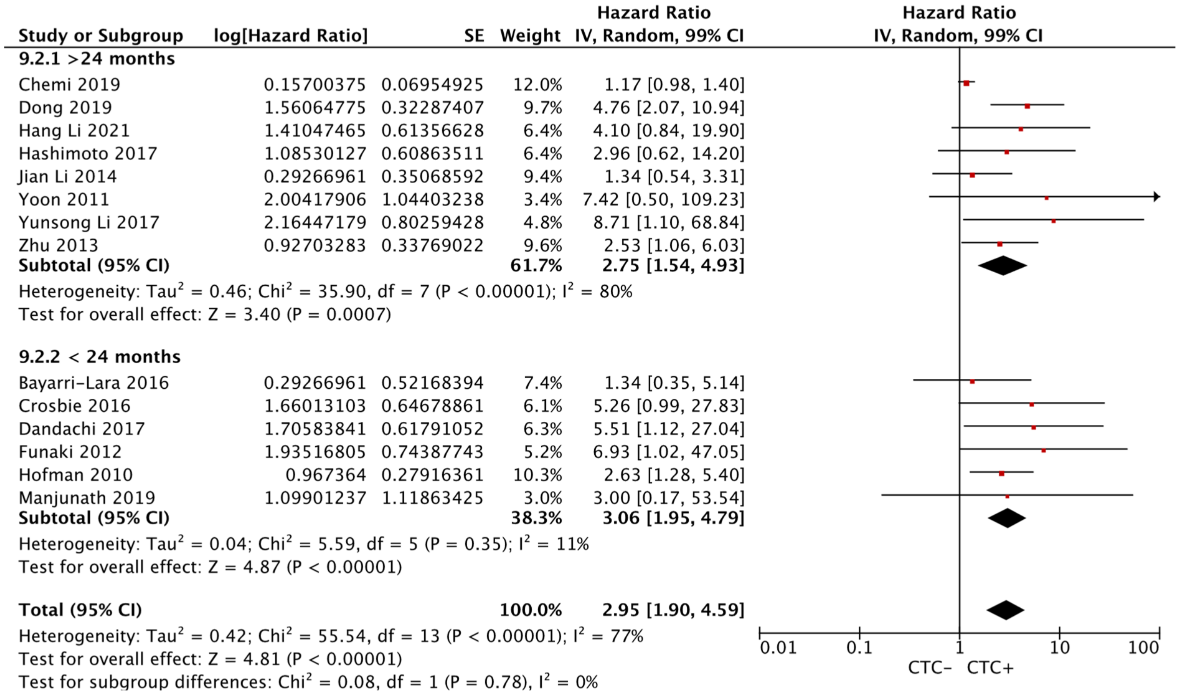

Figure S5. Publication bias : Funnel plot for primary analysis

A. Overall survival

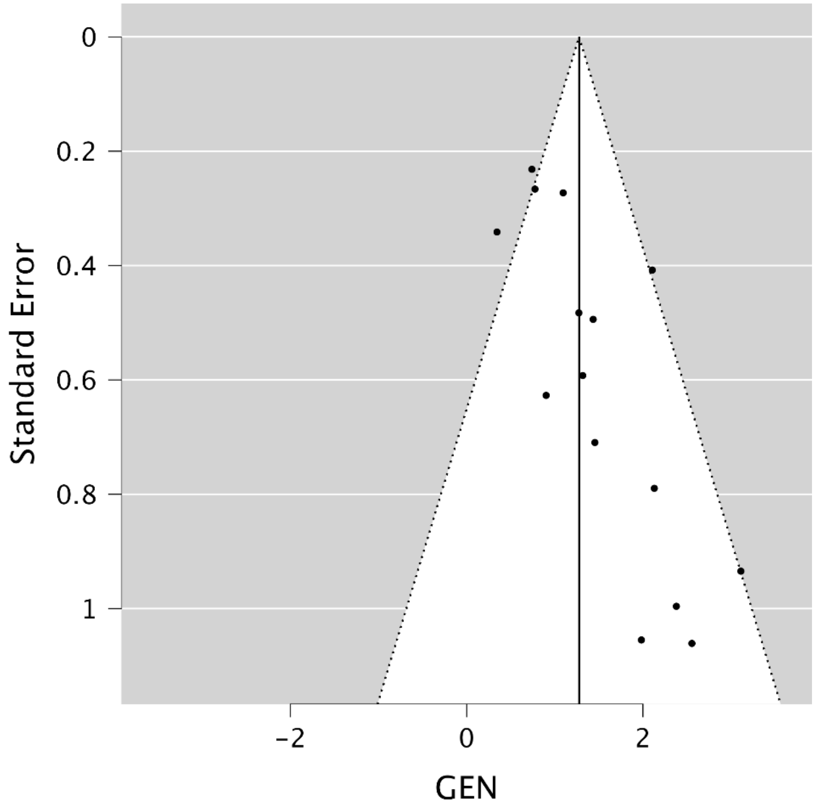

B. Disease-Free Survival

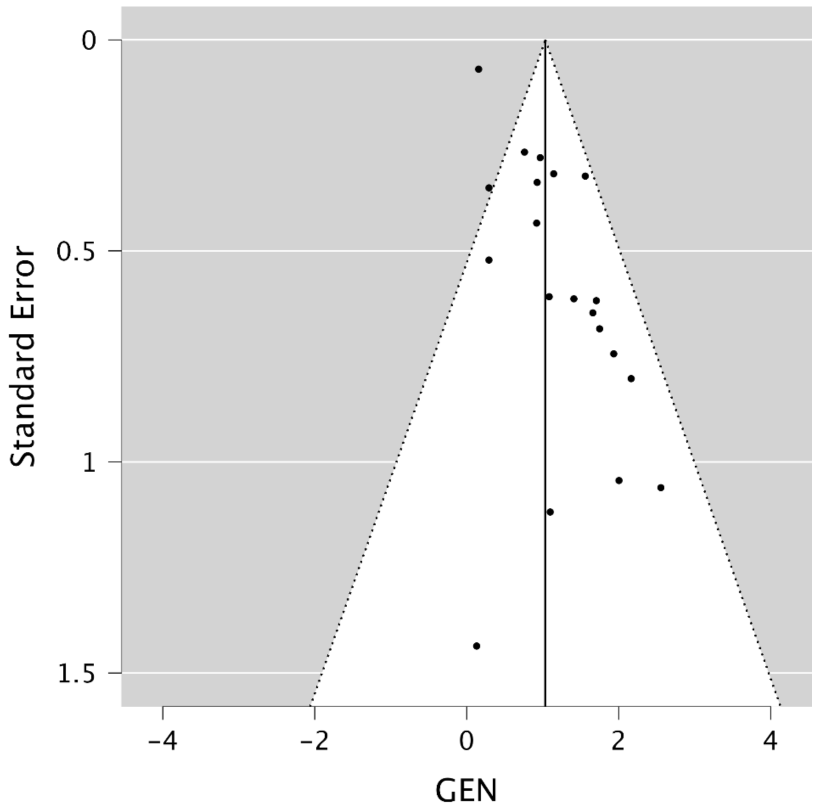

Figure S6. Publication bias: Trim-and-fill method

A. Overall Survival

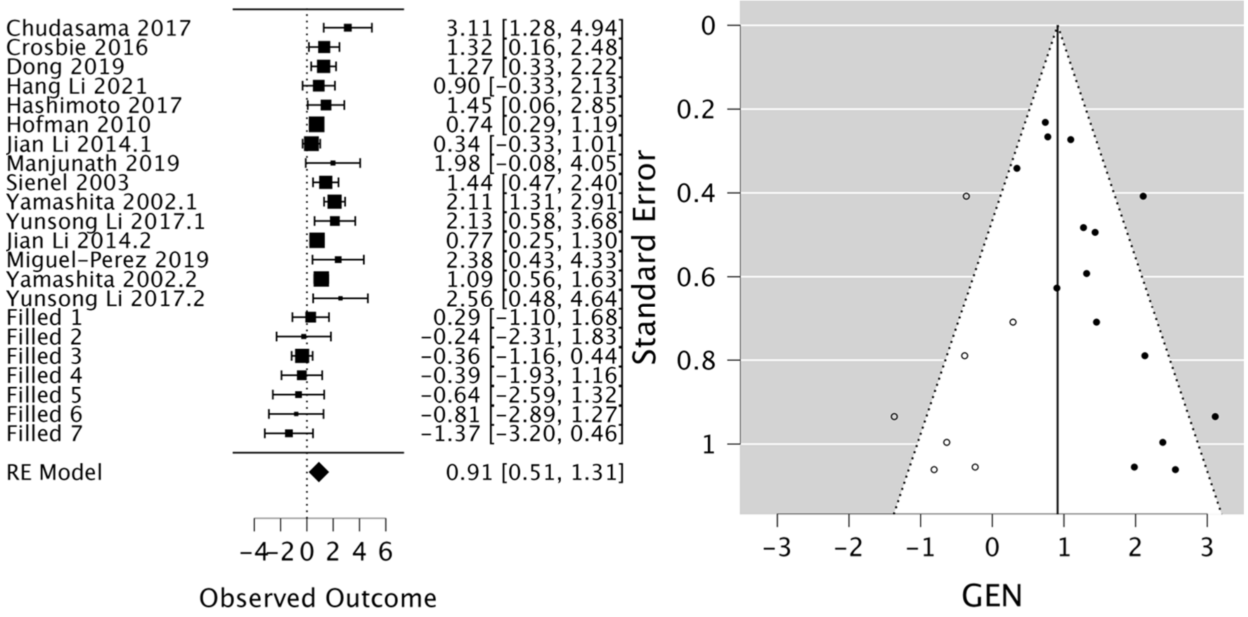

B. Disease-Free Survival

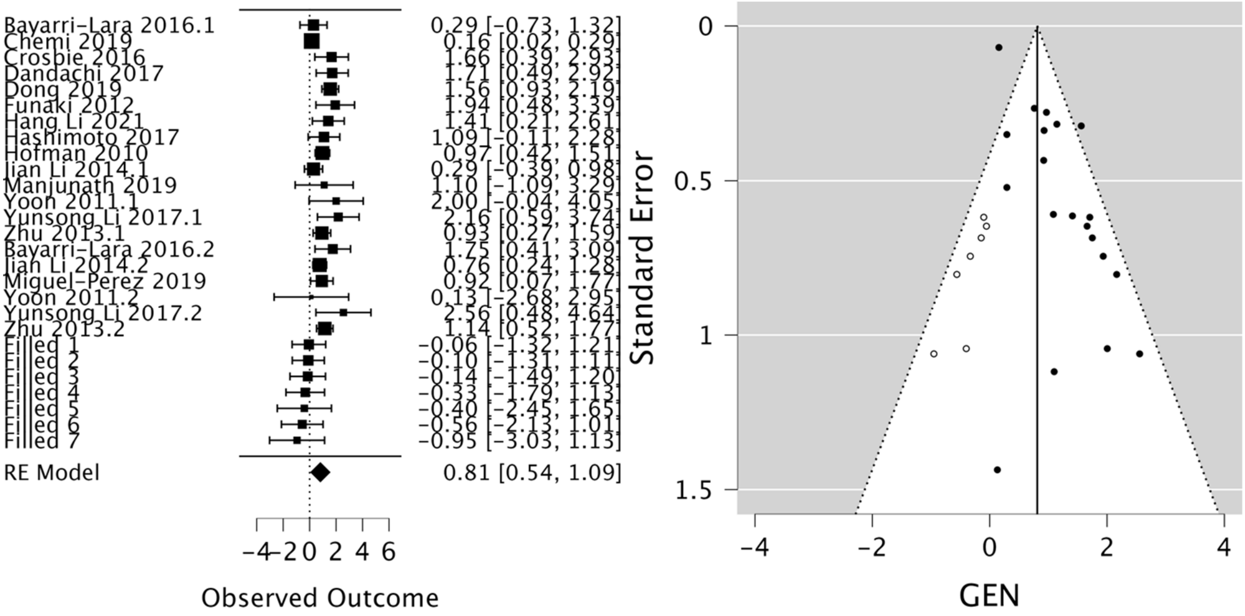

Figure S7. Correlation of clinicopathologic characteristics with CTC

A. Male gender:

Study or Subgroup	CTC+ Events	CTC+ Total	CTC− Events	CTC− Total	Weight	Odds Ratio M-H, Random, 95% CI
Bayarri-Lara 2016	27	29	23	27	2.4%	2.35 [0.39, 14.01]
Chemi 2019	28	48	33	52	12.0%	0.81 [0.36, 1.80]
Crosbie 2016	4	7	12	23	2.7%	1.22 [0.22, 6.73]
Funaki 2012	26	53	22	34	9.9%	0.53 [0.22, 1.27]
Hang Li 2021	76	170	73	177	42.9%	1.15 [0.75, 1.76]
Hashimoto 2017	6	9	12	21	2.9%	1.50 [0.29, 7.68]
Jian Li 2014	29	40	18	28	7.2%	1.46 [0.52, 4.14]
Sienel 2003	8	11	37	51	3.6%	1.01 [0.23, 4.36]
Yoon 2011	14	26	22	35	7.3%	0.69 [0.25, 1.93]
Yunsong Li 2017	6	10	9	13	2.6%	0.67 [0.12, 3.75]
Zhu 2013	17	24	23	37	6.4%	1.48 [0.49, 4.45]
**Total (95% CI)**		**427**		**498**	**100.0%**	**1.02 [0.78, 1.35]**
Total events	241		284			

Heterogeneity: $Tau^2 = 0.00$; $Chi^2 = 5.58$, df = 10 (P = 0.85); $I^2 = 0\%$
Test for overall effect: Z = 0.17 (P = 0.87)

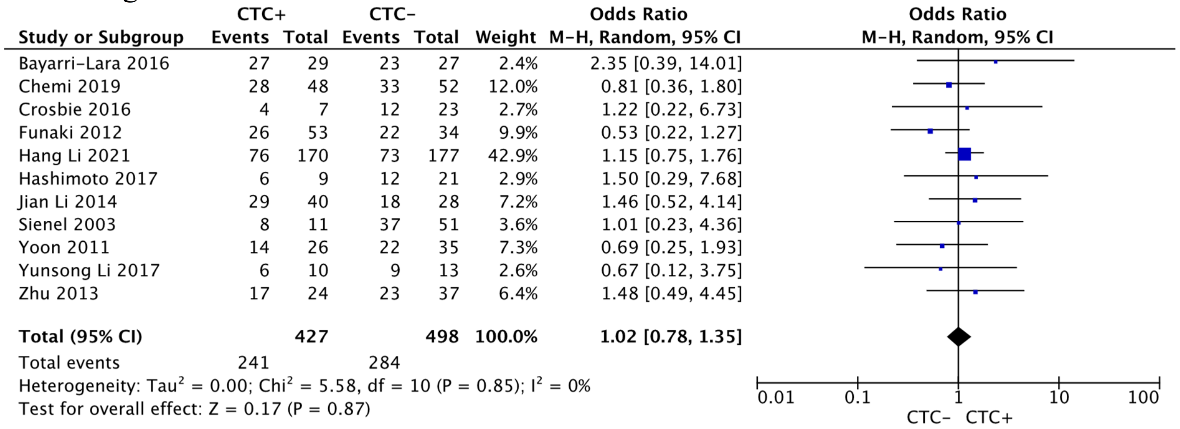

B. Adenocarcinoma

Study or Subgroup	CTC+ Events	CTC+ Total	CTC− Events	CTC− Total	Weight	Odds Ratio M-H, Random, 95% CI
Bayarri-Lara 2016	11	29	14	27	10.3%	0.57 [0.20, 1.64]
Chemi 2019	28	48	31	52	12.6%	0.95 [0.43, 2.11]
Crosbie 2016	1	7	7	23	4.2%	0.38 [0.04, 3.78]
Funaki 2012	41	53	21	34	11.3%	2.12 [0.82, 5.44]
Hashimoto 2017	5	9	17	21	6.3%	0.29 [0.05, 1.62]
Hofman 2010	27	42	93	168	13.5%	1.45 [0.72, 2.93]
Jian Li 2014	31	40	9	28	10.2%	7.27 [2.45, 21.54]
Sienel 2003	1	11	18	51	4.6%	0.18 [0.02, 1.55]
Yoon 2011	13	26	13	35	10.6%	1.69 [0.60, 4.74]
Yunsong Li 2017	7	10	4	13	5.9%	5.25 [0.87, 31.55]
Zhu 2013	15	24	20	37	10.5%	1.42 [0.50, 4.05]
**Total (95% CI)**		**299**		**489**	**100.0%**	**1.30 [0.76, 2.23]**
Total events	180		247			

Heterogeneity: $Tau^2 = 0.43$; $Chi^2 = 23.43$, df = 10 (P = 0.009); $I^2 = 57\%$
Test for overall effect: Z = 0.97 (P = 0.33)

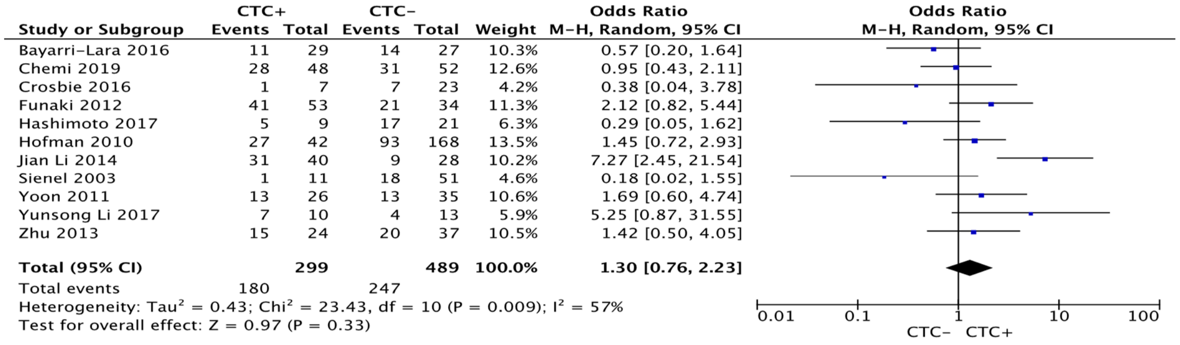

C. Pathologic stage I

Study or Subgroup	CTC+ Events	CTC+ Total	CTC− Events	CTC− Total	Weight	Odds Ratio M-H, Random, 95% CI
Bayarri-Lara 2016	13	29	13	27	9.6%	0.88 [0.31, 2.50]
Chemi 2019	22	48	25	52	13.6%	0.91 [0.42, 2.01]
Crosbie 2016	1	7	11	23	2.8%	0.18 [0.02, 1.76]
Funaki 2012	42	53	30	34	7.6%	0.51 [0.15, 1.75]
Hang Li 2021	123	170	145	177	19.7%	0.58 [0.35, 0.96]
Hashimoto 2017	5	9	17	21	4.5%	0.29 [0.05, 1.62]
Hofman 2010	21	42	65	168	15.7%	1.58 [0.80, 3.13]
Jian Li 2014	5	40	9	28	7.7%	0.30 [0.09, 1.03]
Yoon 2011	13	26	20	35	10.0%	0.75 [0.27, 2.08]
Yunsong Li 2017	1	10	7	13	2.6%	0.10 [0.01, 0.98]
Zhu 2013	3	24	12	37	6.3%	0.30 [0.07, 1.20]
**Total (95% CI)**		**458**		**615**	**100.0%**	**0.62 [0.42, 0.93]**
Total events	249		354			

Heterogeneity: $Tau^2 = 0.14$; $Chi^2 = 15.36$, df = 10 (P = 0.12); $I^2 = 35\%$
Test for overall effect: Z = 2.34 (P = 0.02)

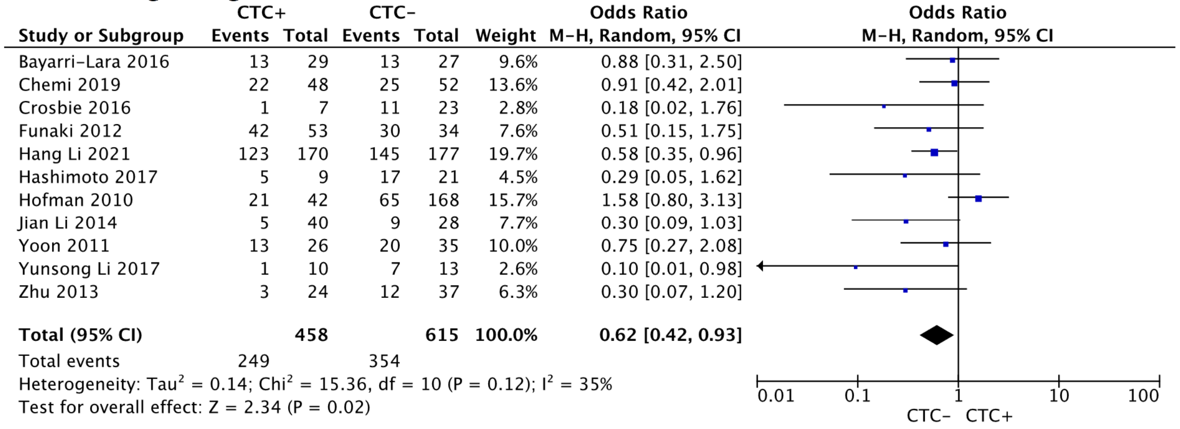

D. Pathologic stage II

Study or Subgroup	CTC+ Events	CTC+ Total	CTC− Events	CTC− Total	Weight	Odds Ratio M-H, Random, 95% CI
Bayarri-Lara 2016	16	29	14	27		Not estimable
Chemi 2019	15	48	19	52	14.4%	0.79 [0.34, 1.81]
Crosbie 2016	5	7	6	23	4.7%	7.08 [1.07, 46.68]
Funaki 2012	5	53	3	34	6.8%	1.08 [0.24, 4.83]
Hang Li 2021	15	170	13	177	15.5%	1.22 [0.56, 2.65]
Hashimoto 2017	6	9	7	21	5.8%	4.00 [0.76, 20.96]
Hofman 2010	16	42	35	168	16.4%	2.34 [1.13, 4.83]
Jian Li 2014	21	40	15	28	12.3%	0.96 [0.36, 2.52]
Yoon 2011	8	26	7	35	9.6%	1.78 [0.55, 5.75]
Yunsong Li 2017	1	10	6	13	3.2%	0.13 [0.01, 1.34]
Zhu 2013	10	24	18	37	11.3%	0.75 [0.27, 2.13]
**Total (95% CI)**		**429**		**588**	**100.0%**	**1.30 [0.83, 2.04]**
Total events	102		129			

Heterogeneity: $Tau^2 = 0.18$; $Chi^2 = 14.34$, df = 9 (P = 0.11); $I^2 = 37\%$
Test for overall effect: Z = 1.16 (P = 0.24)

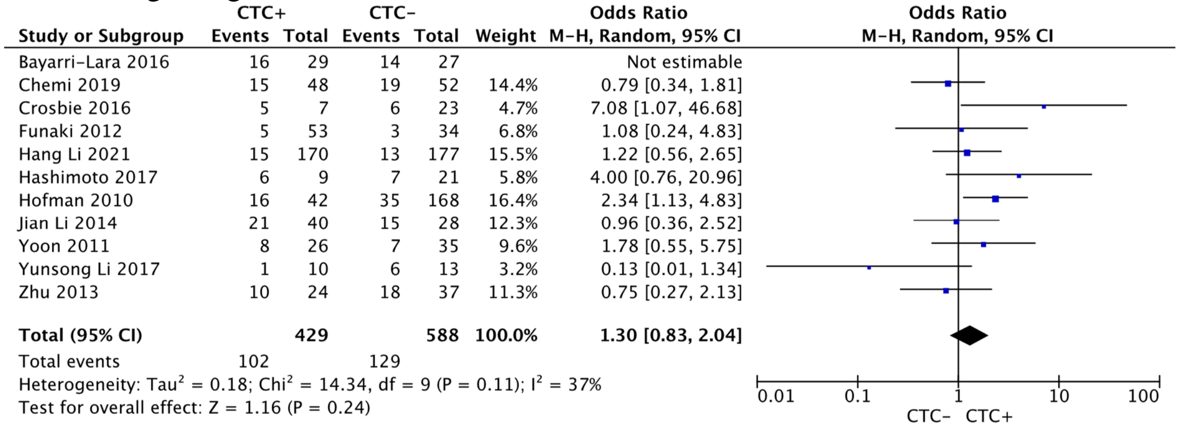

E. Pathologic stage III

Study or Subgroup	CTC+ Events	CTC+ Total	CTC− Events	CTC− Total	Weight	Odds Ratio M-H, Random, 95% CI
Chemi 2019	11	48	8	52	15.2%	1.64 [0.60, 4.49]
Crosbie 2016	1	7	6	23	4.9%	0.47 [0.05, 4.77]
Funaki 2012	6	53	1	34	5.5%	4.21 [0.48, 36.65]
Hang Li 2021	18	170	7	177	17.0%	2.88 [1.17, 7.07]
Hofman 2010	20	42	38	168	20.4%	3.11 [1.54, 6.30]
Jian Li 2014	14	40	2	28	8.9%	7.00 [1.44, 33.92]
Yoon 2011	5	26	8	35	12.0%	0.80 [0.23, 2.82]
Yunsong Li 2017	8	10	0	13	2.9%	91.80 [3.91, 2153.66]
Zhu 2013	11	24	7	37	13.3%	3.63 [1.15, 11.45]
**Total (95% CI)**		**420**		**567**	**100.0%**	**2.65 [1.51, 4.64]**
Total events	94		77			

Heterogeneity: $Tau^2 = 0.27$; $Chi^2 = 13.55$, df = 8 (P = 0.09); $I^2 = 41\%$
Test for overall effect: Z = 3.41 (P = 0.0007)

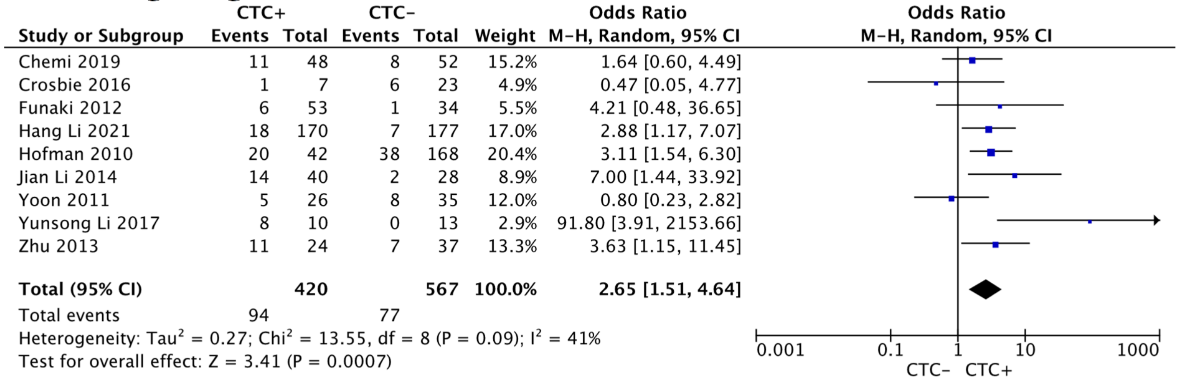

Supplement: Supplementary file 1 [file cancers-14-06112-s001.zip › cancers-2045502-supplementary.pdf]
